# The global regulator Ncb2 escapes from the core promoter and impacts transcription in response to drug stress in *Candida albicans*

**DOI:** 10.1038/srep46084

**Published:** 2017-04-06

**Authors:** Mohd Shariq, Sanjiveeni Dhamgaye, Remya Nair, Neha Goyal, Vaibhav Jain, Arnab Mukhopadhyay, Alok K. Mondal, Gauranga Mukhopadhyay, Rajendra Prasad

**Affiliations:** 1 School of Life Sciences, Jawaharlal Nehru University, New Delhi-110067, India; 2 Special Centre for Molecular Medicine, Jawaharlal Nehru University, New Delhi-110067, India; 3 Department of Microbiology, Monash University, VIC, Australia; 4 Rajiv Gandhi Institute of I.T and Biotechnology, Bharati Vidyapeeth University, Pune-411045, India; 5 Molecular Aging Laboratory, National Institute of Immunology, Aruna Asaf Ali Marg, New Delhi-110067, India; 6 Amity Institute of Integrative Sciences and Health and Amity Institute of Biotechnology, Amity University, Haryana-122413, India

## Abstract

Ncb2, the β subunit of NC2 complex, a heterodimeric regulator of transcription was earlier shown to be involved in the activated transcription of *CDR1* gene in azole resistant isolate (AR) of *Candida albicans*. This study examines its genome-wide role by profiling Ncb2 occupancy between genetically matched pair of azole sensitive (AS) and AR clinical isolates. A comparison of Ncb2 recruitment between the two isolates displayed that 29 genes had higher promoter occupancy of Ncb2 in the AR isolate. Additionally, a host of genes exhibited exclusive occupancy of Ncb2 at promoters of either AR or AS isolate. The analysis also divulged new actors of multi-drug resistance, whose transcription was activated owing to the differential occupancy of Ncb2. The conditional, sequence-specific positional escape of Ncb2 from the core promoter in AS isolate and its preferential recruitment to the core promoter of certain genes in AR isolates was most noteworthy means of transcription regulation. Together, we show that positional rearrangement of Ncb2 resulting in either activation or repression of gene expression in response to drug-induced stress, represents a novel regulatory mechanism that opens new opportunities for therapeutic intervention to prevent development of drug tolerance in *C. albicans* cells.

The commensal *Candida albicans* turns into a life threatening pathogen in immunocompromised individuals. For this lethality, *C. albicans* has an array of important traits, including yeast to hyphae transition, secretion of adhesion molecules and phenotypic switching^1^,^2^,^3^. Candidiasis caused by *C. albicans* is the fourth most common infections in hospitals which on several occasions are systemic and fatal^4^,^5^,^6^. Most commonly used antifungal drugs target unique components of fungal cells, which include either plasma membrane or cell wall (CW). For example, polyenes and azoles target ergosterol, a major unique component of fungal cell membrane while echinocandins target typical CW components. Fluconazole (FLC) is the most common azole which is widely used in clinics due to its low toxicity and easy oral administration^7^. However, the prolonged use of fungistatic FLC results in the development of tolerance towards the drug.

The *Candida* cells employ several strategies to acquire resistance not only to the drug in use but also display collateral resistance to other unrelated drugs. Among various mechanisms which contribute in acquiring multi-drug resistance (MDR), enhanced drug export represents an important strategy adopted by the pathogen^8^,^9^,^10^. Thus, most of the clinical MDR isolates of *C. albicans* are shown to over-express *CDR1, CDR2* or *MDR1* drug efflux pump protein-encoding genes. Cdr1 and Cdr2 belong to the ATP binding cassette (ABC) super-family of transporter proteins and use energy derived from ATP hydrolysis to expel drugs to the cell exterior. Mdr1 on the other hand is an H^+^/drug antiporter which belongs to the super-family of major facilitators (MFS)^11^,^12^,^13^. Notably, both ABC and MFS family of transporters contribute to MDR in *Candida*; the regulatory circuits which control their gene expression are different^13^,^14^,^15^.

*CDR1* is a major drug efflux protein-encoding gene which is regulated at the transcriptional as well as at post transcriptional levels^9^,^16^,^17^,^18^,^19^,^20^,^21^,^22^. It has been shown that *CDR1* promoter harbours various cis-regulatory elements (Sp1, AP-1, and Y-box) as well as specific cis-elements such as Basal Regulatory Element (BRE), Negative Regulatory Element (NRE) and Drug/Steroid Response Element (DRE and SRE) in the 5′ flanking region^17^,^20^,^23^,^24^. Trans-acting factors regulating *CDR1* have also been identified. For example, Non dityrosine 80 (Ndt80), a homolog to a meiosis-specific transcription factor (TF) in *Saccharomyces cerevisiae* is an activator of *CDR1*^18^. A regulator was identified to be the Transcriptional Activator of *CDR* genes (Tac1) binds to the Drug Response Element (DRE) in both the *CDR1* and *CDR2* promoters^19^. Interestingly, the zinc cluster family TF Fcr1 (Fluconazole Resistance 1) and the global repressor Tup1 (Thymidine Uptake 1) act as negative regulators of *CDR1* expression^25^,^26^. A genome-wide profiling (ChIP-on-chip) has shown that another TF of the zinc cluster family, Uptake control 2 (Upc2) regulating transcription of the *ERG* genes, also targets *CDR1*^27^.

In addition to the gene-specific transcription factors, there are a variety of accessory factors that act in global manner in regulating MDR genes. For example, chromatin-modifying enzymes and proteins positively or negatively affect the formation of an active transcription initiation complex^28^,^29^,^30^. Negative Cofactor 2 (NC2) is an example of one such global regulator of RNA polymerase II (Pol II) which executes both negative and positive roles in transcription^31^,^32^,^33^,^34^,^35^,^36^. NC2 is a heterodimer comprised of two subunits, NC2α and NC2β (Ncb2) which was originally recognized as a TATA-binding protein (TBP) activity in human nuclear extracts that repressed RNA Pol II transcription^37^,^38^. It was later identified in *S. cerevisiae* as an essential factor and the β and α-subunit-encoding genes are termed as *NCB2 (YDR1*) and *BUR6*, respectively^38^,^39^. NC2 together with Mot1 [(Modifier of transcription 1 (Mot1) is a conserved and essential Swi2/Snf2 ATPase)] and TBP forms evolutionary conserved complex at the promoter regions^40^. It occupies opposite binding surfaces to MOT1 on TBP and encircle DNA bound TBP molecule which recognizes the minor groove of the TATA-sequence^40^. Additionally, NC2 can localize TBP onto the promoters by using its histone fold domain and bind to the underside of the DNA by using C-terminal helix of NC2β which surrounds DNA and binds convex side of TBP^40^,^41^. Consequently, NC2 together with TBP can move laterally on DNA. It has been shown that NC2 induces dynamic conformational changes in the TBP-DNA interface that enables TBP to laterally move on the DNA substrate^40^,^42^.

In an earlier report, we have shown the importance of the β-subunit of NC2 complex in the basal and activated transcription of *CDR1* in azole sensitive (AS-Gu4) and azole resistant (AR-Gu5) isolates of *C. albicans* and also provided evidence that this cofactor may also be involved in the regulation of select genes, particularly those targeted by Tac1^43^. This study evaluates the genome-wide occupancy of Ncb2 between genetically matched, AS and AR isolates of *C. albicans*. The study shows that Ncb2 occupies the promoters of 25–30% of *C. albicans* genes of almost every metabolic class in AR and AS isolates, thereby reinforcing its status as a global regulator of gene expression. Notably, there are hosts of novel MDR genes, which are regulated by Ncb2 in response to drug stress, leading to transcriptional activation.

## Results

### Ncb2 emerged as a global regulator of gene expression in AS and AR isolates of *C. albicans*

**Figure 1:**
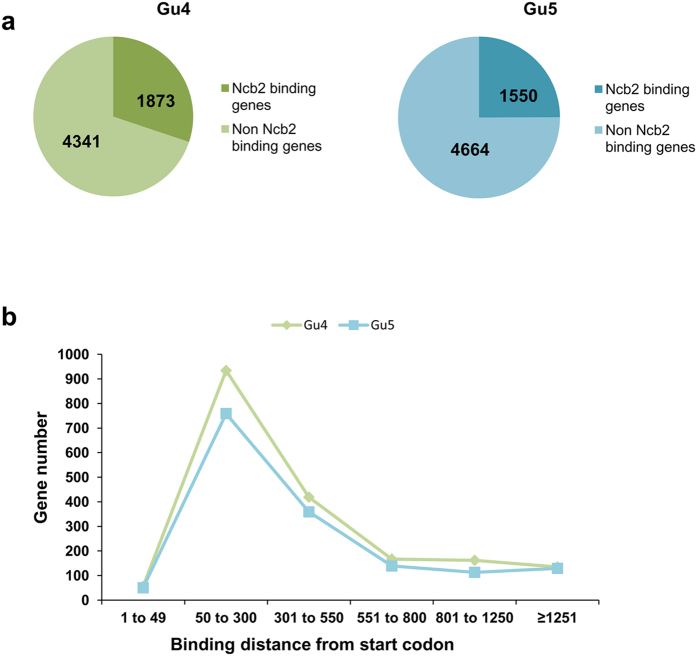
Genome-wide promoter association of Ncb2. (**a**) Charts showing the Ncb2 occupying and non-occupying gene promoters of *C. albicans* in an Ncb2 ChIP performed in Gu4 (AS) and Gu5 (AR) clinical isolates. (**b**) Ncb2 binding peaks localizes at the −50/−300 region encompassing the core promoter. Average binding profiles of Ncb2 for the promoter regions were determined relative to ATG start codon for both the isolates. Green line represents localization profile of Ncb2 in the AS isolate; whereas blue line represents localization profile of Ncb2 in AR isolate. Binding distance was calculated for the probe that has maximum normalized log ratio.

Ncb2, a part of the heterodimeric NC2 complex that controls transcription of genes was earlier shown to be involved in the activated transcription of *CDR1* gene in azole resistant (AR) clinical isolate of *C. albicans*^43^,^44^. To understand the global impact of Ncb2 in acquisition of MDR in *C. albicans*, we performed a genome-wide recruitment analysis of the transcriptional regulator by employing ChIP-on-chip assay and compared it between azole sensitive (AS) and AR isolates. Three biological replicates were processed for each of the isolate to identify DNA regions that were occupied by Ncb2. By using polyclonal anti-Ncb2 antibody, the cross-linked protein-DNA complexes were pulled down^43^. Signal peaks detected in all the three experiments were chosen. Analysis of the genome-wide data revealed no major difference in terms of percentage occupancy of Ncb2 and varied between 25–30% in AR and AS isolates (Fig. 1a). For further analysis, by employing three different arbitrary cutoffs, all the promoters were ranked according to the *P*-values and fold enrichments (in comparison with input DNA); I) genes with *P*-values ≤ 0.055 and fold enrichments ≥1.5 (AS-1873 genes, AR-1550 genes), II) genes with *P*-values ≤ 0.055 and fold enrichments ≥2.0 (AS-1765 genes, AR-1546), III) genes with *P*-values ≤ 0.01 and fold enrichments ≥2.0 (AS-1119, AR-960). Furthermore, to compare the genomic binding of Ncb2 between the isolates, the average binding profile for the promoter (fragments: −1/−49, −50/−300, −301/−550, −551/−800, −801/−1250 and ≥1251, relative to the start codon) was computed which showed that Ncb2 mainly recruited at the core promoter regions^43^,^45^,^46^ (Fig. 1b). Genome-wide occupancy data analysis also revealed that Ncb2 binds both inside and downstream of the genes (Supplementary Figs S1 and S2). The most common model for transcriptional activation is that the binding, just upstream of the transcription start site (TSS) helps in placing polymerase II at the beginning of the gene. Presence of TF binding sites inside or downstream of gene may act as regulatory sequence for the same gene or act as enhancer for the control of same gene or downstream gene (acting as a distal enhancer) in orientation and position independent manner^47^,^48^,^49^. Notably, all binding sites may not be functional or represents a non productive binding site or could just be alternative TSS^50^,^51^,^52^.

### Ncb2 occupies genes with diverse metabolic functions

To get an insight into the functional features of the biological processes that are modulated by Ncb2, we have chosen most stringent arbitrary condition for both the isolates (genes with *P*-values ≤ 0.01 and fold enrichments ≥2.0), and carried out a search at Candida Genome Database (CGD). A closer look at Ncb2 occupancy revealed its binding to the diverse functional classes of genes involved in metabolism, transport, cell wall, filamentous growth, virulence, mitochondrial function, signal transduction, biofilm formation and oxidative stress (Supplementary Tables S1 and S2). Notably, Ncb2 occupancy at the promoter regions of the genes encoding membrane transporter was very conspicuous (Supplementary Tables S1 and S2). For example, Ncb2 not only showed binding at the promoter region of *CDR1* but also at the promoters of ABC transporter genes *CDR2, CDR4* and *MDL2* and MFS transporters including *MDR1, FLU1, NGT1, HGT1, HGT2, HGT7, HGT19, HGT20, ITR1, TPO3* and *RTA2* (Supplementary Tables S1 and S2). Moreover, promoters of well-known MDR determinants like *PDR16, ERG11, NCP1, ERG251* and *ERG9* were also found to be occupied by Ncb2 (Supplementary Tables S1 and S2).

### The higher occupancy of Ncb2 in AR isolate has dual effect on transcription

**Figure 2:**
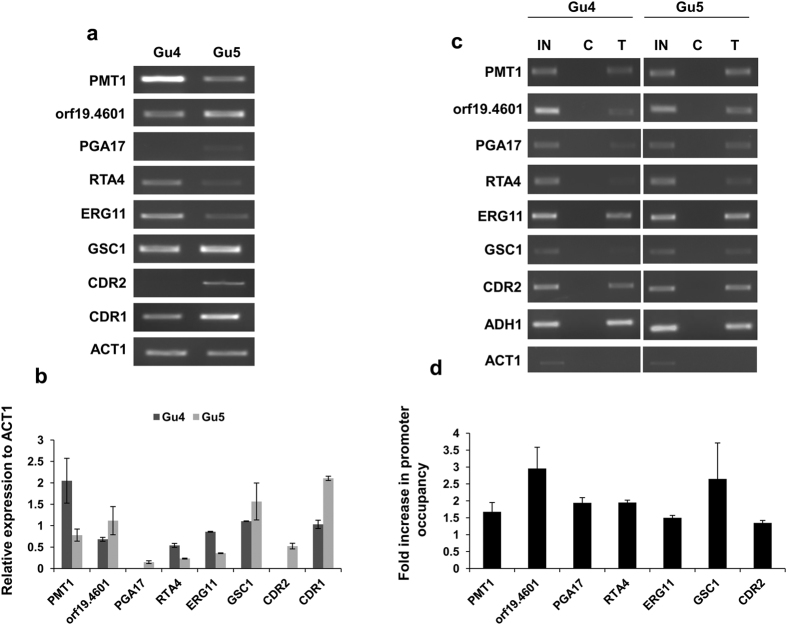
Confirmation of the relation of Ncb2 higher occupancy and gene expression in AR isolate by semi-quantitative RT-PCR and ChIP-PCR. (**a**) End point semi-quantitative RT-PCR was used to validate some of the genes found to be highly enriched by Ncb2 in the AR isolate and their relation to gene expression. RT-PCR was performed in triplicate and a representative figure (cropped gel pictures) is shown. For full gel see Supplementary Fig. S3. *CDR1* and *CDR2* genes were used as positive controls for the gene over-expressed in the AR isolate. *ACT1* expression level was used as a control for isoexpression between AR and AS isolates. (**b**) Bar diagram showing the expression profile of genes highly enriched in AR isolate as compared to AS isolate by Ncb2. Bars represent the standard deviations observed for the replicate experiments. (**c**) Recruitment profiles of Ncb2 in AR isolate as compared to AS isolate determined by ChIP-PCR. For presentation cropped gels are shown. For complete gel see Supplementary Fig. S4. IN represents amplification observed in input sample, control (C) and test (T) are immunoprecipitations carried out on cross-linked chromatin using pre-immune serum and anti-Ncb2 antibody, respectively. Amplification of *CDR2* promoter region was used as a positive control. *ADH1* and *ACT1* promoter regions amplification were used as positive and negative control for Ncb2 binding. (**d**) Bar diagram representing the Ncb2 enrichments of AR over AS isolate. Bars represent standard deviations for the replicate experiments.

We compared the genome-wide recruitment data of Ncb2 between AS and AR isolates and thereby, identified genes that showed distinct occupancy of Ncb2 at the promoter regions. The analysis revealed that a number of genes have higher enrichment of Ncb2 occupancy in AR isolate (Supplementary Table S3). This included genes implicated in drug response, filamentous growth, various stresses, pathogenesis, transport, cell wall organization, signal transduction, and biofilm formation (Supplementary Table S4). To establish a relation between the higher occupancy of Ncb2 in AR isolate and gene expression, we randomly selected 6 genes (*PMT1, orf19.4601, PGA17, RTA4, ERG11* and *GSC1*) of interest with different normalized log ratios (enrichments) and *P*-values. Their expression was checked by semi-quantitative end-point RT (reverse transcription) PCR. *CDR1* and *CDR2* were used as positive controls. Inspite of the higher Ncb2 occupancy, the expression data showed a mixed response of gene expression. For instance, three genes [*orf19.4601* (Putative RNA polymerase III transcription initiation factor complex (TFIIIC) subunit), *PGA17* (Putative GPI-anchored protein) and *GSC1*)] were up-regulated in AR isolate whereas *PMT1* (Protein mannosyltransferase), *RTA4* (Protein similar to *S. cerevisiae* Rsb1p, involved in fatty acid transport) and *ERG11* were down regulated in AR cells (Fig. 2a,b; Supplementary Fig. S3). The validation though limited, points that the higher occupancy of Ncb2 in AR isolate does not always result in transcriptional activation. Nevertheless, Ncb2 probably manifests dual effect on transcriptional control mechanisms. Since genes that showing higher enrichment in ChIP-on-chip data displayed differential gene expression pattern in AS and AR isolates hence we analyzed the relative recruitment dynamics of Ncb2 in both the isolates. For this, we used ChIPed DNA from AS and AR isolates and subjected to ChIP-PCR analysis which revealed that Ncb2 binding correlates well with the ChIP-on-chip data (Fig. 2c,d; Supplementary Fig. S4).

### Genes exclusively enriched by Ncb2 in the AR isolate mostly exhibit activated transcription

**Figure 3:**
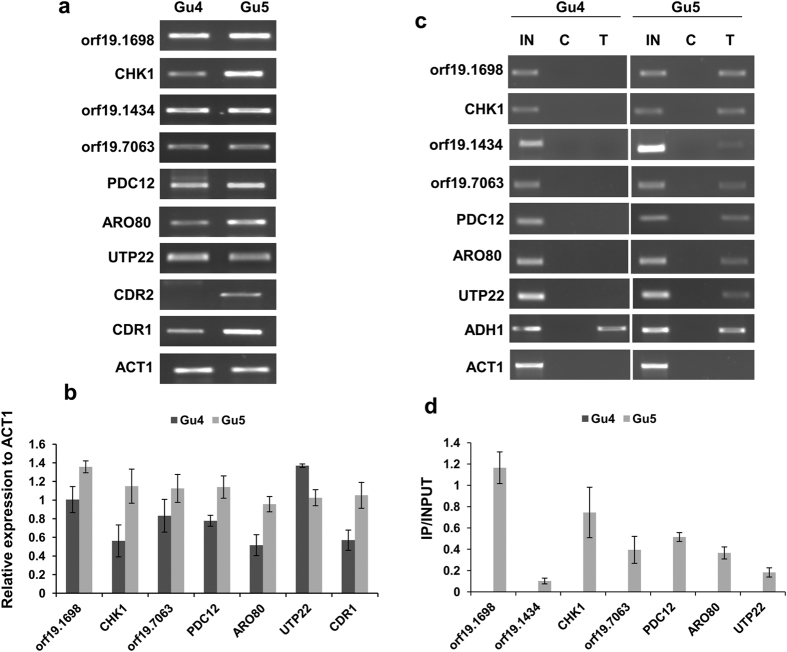
Ncb2 exclusive occupancy in the AR isolate mostly leads to activated transcription of the target genes. (**a**) Semi-quantitative RT-PCR was used to study the effect of exclusive enrichment of Ncb2 and gene expression of selected genes in the AR isolate. Most of the exclusive target genes of Ncb2 in the AR isolate were found activated except for *UTP22* which was repressed. *CDR1* and *CDR2* genes served as controls that over-expressed in the AR isolate. *ACT1* expression level was used as control that equally expresses in both the isolates. RT-PCR was performed from c-DNA prepared from three different RNA preparations. For presentation cropped gels are shown. For complete gel see Supplementary Fig. S5. (**b**) Bar diagram demonstrating expression profile of AR exclusive enriched genes. Bars represent standard deviations observed for the replicate experiments. (**c**) ChIP-PCR analysis of Ncb2 exclusive occupancy in AR isolate. Chromatin immunoprecipitation was performed on cross linked chromatin isolated from AS and AR isolates, respectively. Control and test designate immunoprecipitations performed on cross-linked chromatin with pre-immune serum and anti-Ncb2 antibody, respectively. Amplification of *ADH1* and *ACT1* promoter regions were used as a positive and negative control for Ncb2 binding in both the isolates. Cropped gels are used for presentation, for complete gel see Supplementary Fig. S6. (**d**) Bar diagram showing the Ncb2 exclusive occupancy in AR isolate as compared to AS isolate. Bars represent the standard deviations observed for the replicate experiments.

The analysis of the genome-wide Ncb2 recruitment dynamics revealed 25 gene promoters which displayed an exclusive occupancy of Ncb2 in the AR isolate whereas its binding was absent in AS isolate (Supplementary Table S5). In this category, for validation, we carried out expression analysis for the genes which included *orf19.1698, CHK1, orf19.1434, orf19.7063, PDC12, ARO80* and *UTP22* with different enrichment ratios and *P*-values and performed end point semi-quantitative RT-PCR analysis (Fig. 3a,b; Supplementary Fig. S5). Remarkably, with the exception of *UTP22*, most of the genes showing selective enrichment in the AR isolate were accompanied by an increased expression. This implies that the exclusive enrichment of Ncb2 in AR isolate mainly result in transcriptional activation (Fig. 3a,b; Supplementary Fig. S5). Subsequent ChIP-coupled PCR on ChIPed DNA obtained from AS and AR isolates validated the exclusive recruitment dynamics of Ncb2 (Fig. 3c,d; Supplementary Fig. S6). These results revealed that Ncb2 binding was exclusively present only in the ChIPed DNA isolated from the AR isolate (Fig. 3c,d; Supplementary Fig. S6). Therefore, it can be concluded reasonably that Ncb2 exclusively occupies a subset of genes in the AR isolate and is primarily involved in the positive regulation of gene expression (Fig. 3a,b,c,d; Supplementary Figs S5 and S6).

### Ncb2 acts both as a positive and negative regulator of gene expression in the AS isolate

**Figure 4:**
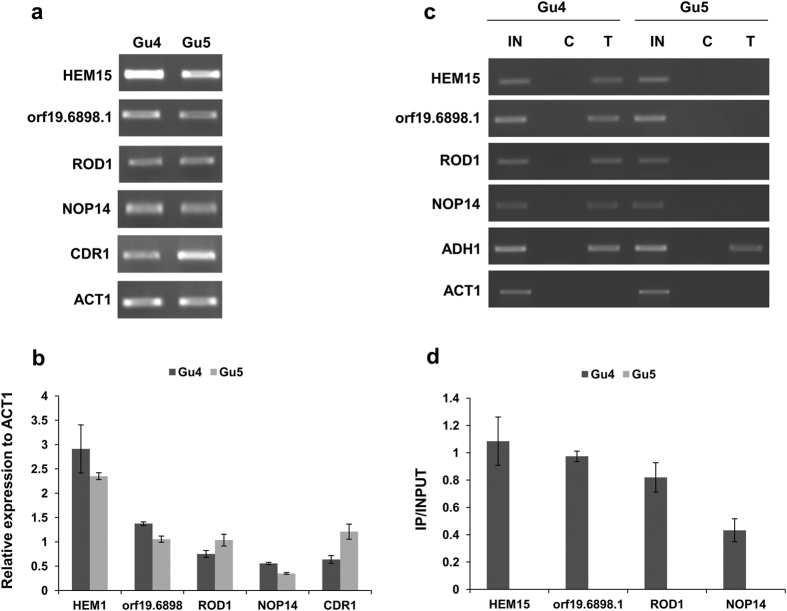
Ncb2 acts as a positive as well as negative regulator of the gene expression of AS exclusive target genes. (**a**) To establish a relation between Ncb2 AS-specific enrichment and gene transcription, we employed semi-quantitative RT-PCR analysis for selected genes. Expression data shows that it acts to control gene expression positively and negatively in the AS isolate. *CDR1* expression level was used as a control for genes induced in AR strain. *ACT1* expression level was used for the normalization of the data. RT-PCR was performed from RNA isolated from three different preparations. Cropped gels were used for presentation. Complete gels are shown in Supplementary Fig. S7. (**b**) Bar diagram was used to find out the differences in the gene expression of genes exclusively occupied by Ncb2 in the AS isolate. Bars represent the standard deviation observed for the replicate experiments. (**c**) ChIP analysis of the promoter regions of selected genes of Ncb2 exclusive occupancy in AS isolate. Control and test represents experiment performed on cross-linked chromatin with pre-immune and anti-Ncb2 antibody. Precipitation of *ADH1* and *ACT1* promoter regions were used as positive and negative control for Ncb2 binding. For presentation purpose cropped gels are shown. For full gel see Supplementary Fig. S8. (**d**) Bar diagram showing the exclusive occupancy of Ncb2 in AS isolate as compared to AR isolate. Bars represent the standard deviation observed for the replicate experiments.

The comparative analysis of the genome-wide binding profiles of Ncb2 in the AR and AS isolates also identified genes whose promoters were exclusively enriched in the AS isolate (Supplementary Table S6). GO slim analysis of the genes exclusively enriched in AS isolate showed that these are involved in transport, filamentous growth, response to drug, pathogenesis, and response to stress etc. (Supplementary Table S7). Four genes were chosen with different binding strength of Ncb2 to establish a relation between exclusive occupancy in AS isolate and gene transcription. The validation of the relation between Ncb2 exclusive occupancy in AS isolate and expression of this group of genes confirmed that the Ncb2 has a dual role both as a positive and negative regulator of gene expression (Fig. 4a,b; Supplementary Fig. S7). Interestingly, ChIP-coupled PCR from the ChIPed DNA obtained from AR and AS isolate of these genes confirmed that Ncb2 only occupies the genes of AS isolate (Fig. 4c,d; Supplementary Fig. S8).

### Ncb2 impacts the transcription of MDR genes

In addition to the global regulatory role of Ncb2, comprehensive analysis of the genome-wide data indicates that it may also have gene-specific functions. For instance, it was observed that Ncb2 differentially occupies promoter regions of the genes that are specifically known to be over-expressed in AR isolates. Among these are the well-known MDR genes *CDR1, CHK1, IFU5, RTA3, CDR2* and *LCB4*, whose over-expression is characteristic of several drug-resistant clinical isolates of *C. albicans*^53^. Several other genes that showed Ncb2 differential enrichment in AS and AR isolates which included *orf19.2204, orf19.1698, orf19.5614, PDC12, orf19.1676, ADAEC*, and *ECE1* was also noteworthy. Activated transcription of these genes is reported in one or two azole-resistant clinical isolates, therefore implying their putative roles in MDR^53^,^54^.

Previous ChIP-on-chip experiments in *C. albicans* identified 37 gene promoters that were occupied by Tac1, a Zn(II)2Cys6 family transcription factor involved in the transcriptional activation of *CDR1* and *CDR2*^9^,^19^,^53^. The Tac1 regulon includes genes involved in the lipid metabolic process, oxidative stress response regulators and genes of unknown functions^53^. Notably, earlier we observed that apart from *CDR1* and *CDR2*, Ncb2 could regulate other Tac1 target genes i.e. *RTA3, IFU5, LCB4, GPX1* and *orf19.1887*^43^. Our present analysis extends this observation to show that Ncb2 is involved in the coordinated transcriptional regulation of most of the Tac1 regulon genes (Supplementary Table S8).

### Ncb2 escapes upstream of the core promoters/TATA-box regions in AS isolate

**Figure 5:**
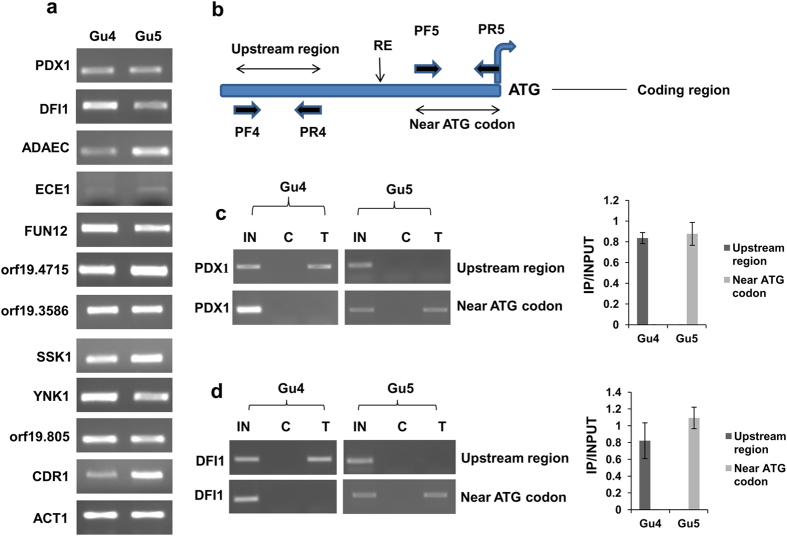
Positional shift at core promoter regions in the AR isolate is not a transcriptional activation mechanism. (**a**) The expression analysis of the selected genes by end point semi-quantitative RT-PCR revealed that Ncb2 acts as activator and repressor of gene transcription in AR isolate. *CDR1* expression level was used as positive control for genes over-expressed in AR isolate. *ACT1* expression level was used as endogenous control for both the isolates. Cropped gel bands are shown, for complete gel see Supplementary Fig. S10. (**b**) Schematic representation of the positions of primer sets used in chromatin restriction digestion couple immunoprecipiation (CRIP) experiments. (**c**) CRIP assay depicting the recruitment of Ncb2 at the core promoter upstream region of *PDX1* gene in AS isolate whereas recruitment was found at core promoter region near ATG codon in AR isolate. IN, C, and T denote input DNA and immunoprecipitation found with control pre-immune serum and anti-Ncb2 antibody, respectively. A bar diagram displaying the difference in occupancy of Ncb2 at core promoter upstream and core promoter near ATG codon containing fragments of the *PDX1* promoter between AR and AS isolates is shown on the right. (**d**) CRIP assay showing the Ncb2 differential position occupancy at *DFI1* promoter upstream region and at core promoter region in AS and AR isolates. Cropped gels are shown, see Supplementary Fig. S11 for full gel. On the right side of the figure a bar diagram representing the difference in occupancy of Ncb2 in AR and AS isolates is shown.

Earlier, we had observed that the recruitment of Ncb2 at the core promoter or TATA-box region led to an activated transcription of *CDR1* in the AR isolate whereas its shift in binding to upstream of the TATA-box region in AS isolate resulted in its repression^43^. It is proposed that NC2 binds and stabilizes TBP to retain the initiation factors at the promoter regions wherein it also exerts dynamic changes in the conformation of TBP-DNA complex and mobilizes TBP on the DNA^42^. Since it is shown that in case of *CDR1*-activated transcription, Ncb2 together with TBP moves on the DNA, we explored if positional shift of Ncb2 is also associated with the regulation of other genes^42^,^43^. We identified 35 genes that display Ncb2 occupancy at the core promoters/TATA-box regions in AR isolate as compared to the AS isolate where it escapes and binds upstream of the core promoters/TATA-box regions (Supplementary Table S9). These genes belong to the biofilm formation (*ZCF8, NDH51, orf19.7459, SUN41 PDX1, CRZ2, ECE1*), filamentous growth (*ZCF8, NDH51, orf19.7459, SUN41, PDX1, SSK1*), response to stress (*NDH51, CTA8, SSK1, CRZ2*), response to drug (*CDR1, EFT2*), translation (*FUN12, EFT2*), transport (*GIT3, CDR1*), cell wall organization (*SUN41, SSK1*), and pathogenesis (*SUN41, SSK1*), as identified by GO slim analysis (Supplementary Table S10). We hypothesized that selective occupancy of Ncb2 at the core promoter regions in the AR isolate may be a transcriptional activation mechanism, like we observed for *CDR1*. However, here the expression data revealed that the positional shift involves both transcriptional activation and repression. Nonetheless, it appears that the positional shift in Ncb2 occupancy for a subset of genes in the presence of drug stress is a novel regulatory mechanism that may either involve positive or negative regulation of gene expression (Fig. 5a; Supplementary Figs S9 and S10). For example, out of 9 genes selected for validation, 4 genes showed over-expression (*orf19.4715, SSK1, ADAEC* and *ECE1*) while 5 genes were repressed (*FUN12, DFI1, YNK1, orf19.805* and *PDX1*) in the AR isolate (Fig. 5a; Supplementary Figs S9 and S10).

**Figure 6:**
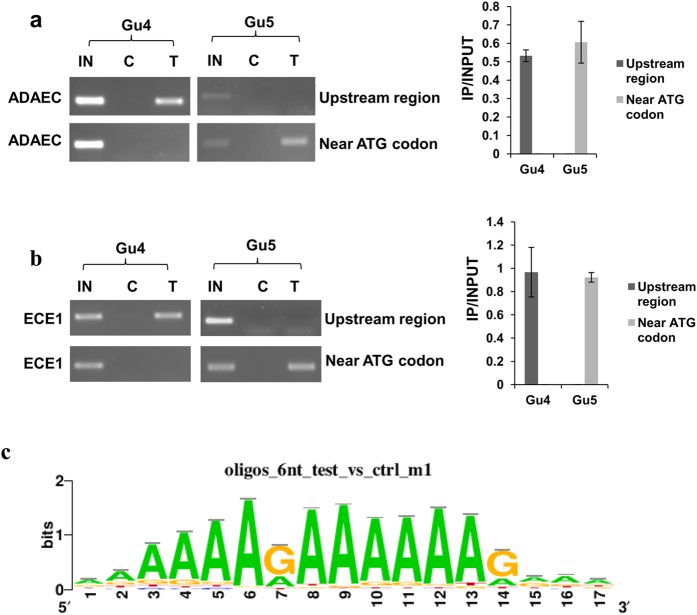
Ncb2 occupies different positions at promoter regions of *ADAEC* and *ECE1* in AS and AR isolates. (**a**) CRIP assay showing the differential recruitment of Ncb2 at *ADAEC* promoter region in AS and AR isolates. The result demonstrate that in AS isolate Ncb2 recruited at the upstream of core promoter region whereas its recruitment was found at the core promoter near ATG codon in AR isolate. On the right side of the figure a bar diagram depicting the difference in enrichment profiles is shown. (**b**) Ncb2 occupy different positions at promoter regions of *ECE1* in AS and AR isolates. CRIP assay showing the differential recruitment profiles of Ncb2 at gene promoter of *ECE1*. Its occupancy was found at upstream region of core promoter in AS isolates whereas it occupies at the core promoter region in AR isolates. On the right side of the figure a bar diagram representing the observed differences of Ncb2 occupancy in AS and AR isolates is shown. For demonstration cropped gel bands are shown. Complete gels are shown in Supplementary Fig. S12. (**c**) Ncb2 preferentially recruited at A-rich sequence motif of DNA. Logo of the Ncb2 DNA binding motif discovered by Rsat using 500 promoter regions that have highest enrichment profiles in ChIP-on-chip data.

Further, shift in the positional occupancy of Ncb2 was confirmed by using chromatin restriction digestion coupled immunoprecipitation (CRIP) assay. For this, genes that displayed shift in occupancy of Ncb2 and showed regulated gene expression in AS and AR isolates were chosen. CRIP assay was performed with genes such as *PDX1, DFI1, ADAEC* and *ECE1* in AS and AR isolates. Their promoter DNA was separated into ATG codon containing core promoter and upstream of the core promoter fragments by using HinFI, EcoRV, XhoI and DpnI site as depicted in Fig. 5b. The fragmented chromatin was precipitated by using anti-Ncb2 antibody followed by PCR using two primer pairs for each gene (Fig. 5b). Interestingly, it was observed that in the AS isolate Ncb2 occupancy was detected at upstream of the core promoter regions while there was no occupancy at the core promoter near ATG codon (Figs 5c,d and 6a,b; Supplementary Figs S11 and S12). Of note, a totally reverse scenario was observed in the AR isolate where Ncb2 was exclusively recruited at the core promoter regions (Figs 5c,d and 6a,b; Supplementary Figs S11 and S12).

**Figure 7:**
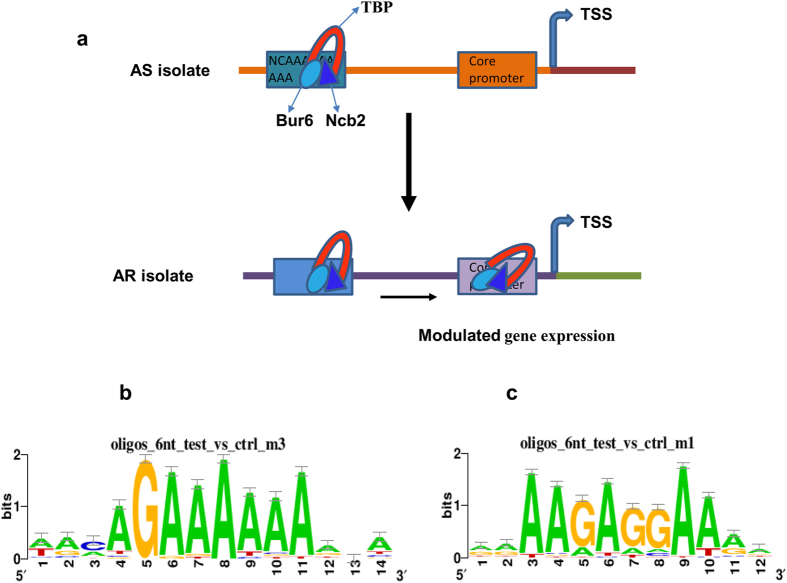
Ncb2 occupies core promoter-TATA-box regions in AR isolate. (**a**) Hypothetical depiction of the preferred positional occupancy of Ncb2 at core promoter or TATA-box regions in a number of genes in AR isolate. TBP and TSS stand for TATA-binding protein and transcription start site, respectively. Bur6 and Ncb2 denote α and β-subunits of NC2 complex. (**b**) Logo demonstrating A-rich sequence motif of Ncb2 binding in the DNA sequence of TATA-upstream regions/upstream of core promoter regions in the AS isolate. (**c**) Illustration of sequence logo of Ncb2 binding motif in the DNA sequences of core promoter/TATA-box regions in the AR isolate.

A hypothetical model depicting differential positional occupancy at the promoter regions is shown in Fig. 7a. Analysis of the promoter regions of upstream positional shift in AS isolate revealed that Ncb2 prefers different positions at the promoter regions of the genes. For instance, the distance of Ncb2 occupancy at A-rich Ncb2 binding motif of DNA varied between −135 bp and −3659 bp (Fig. 7b; Supplementary Table S9). In contrast, in AR isolate Ncb2 occupies the core promoter region at a slightly dissimilar sequence in comparison to AS isolate (Fig. 7c).

### Tac1 plays a role in regulation of AR specific target genes of Ncb2

**Figure 8:**
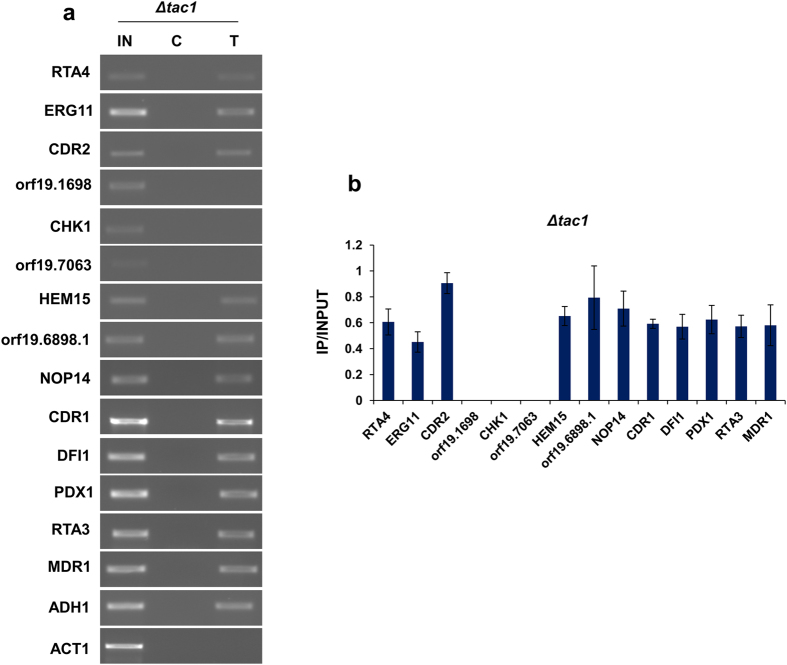
Ncb2 recruitment at the promoters of AR specific genes may be co-regulated with Tac1. (**a**) ChIP assay of Ncb2 recruitment with the promoters of a selected set of MDR genes of *C. albicans* in *Δtac1* strain. Cropped gel pictures are shown for presentation. For complete gel pictures see Supplementary Fig. S13. (**b**) The enrichment of Ncb2 recruitment at the promoters of selected MDR genes in *Δtac1* strain is represented as a bar diagram. Error bars denote standard deviations for the replicate experiments. IN, C, and T are the same as those described for earlier figures.

Earlier, we had evaluated the possible role of Ncb2 in regulating MDR genes, specifically those co-regulated with *CDR1* by the transcription regulator, Tac1. We observed that Ncb2 occupied the promoter regions of a few of the Tac1-regulon genes^43^. In this study, we evaluated whether Ncb2 recruitment is Tac1 dependent. For this, its recruitment dynamics at promoter regions of MDR-genes in the *Δtac1* strain was determined. We observed that Ncb2 was associated at the promoter regions of selected MDR genes from all categories (AR highly enriched genes, AR exclusive genes, AS exclusive genes, positional shift category) which included *RTA4, ERG11, CDR2, HEM15, orf19.6898.1, NOP14, CDR1, DFI1, PDX1, RTA3* and *MDR1*, however, *orf19.1698, CHK1* and *orf19.7063* showed no occupancy of Ncb2. Notably, all the three genes which appeared to be Tac1 dependent belongs to the AR exclusive Ncb2 enriched category. Among control genes included in the analysis, Ncb2 was seen to be bound to the *ADH1* promoter in *Δtac1* strain, while it was not seen on the *ACT1* promoter (Fig. 8a,b; Supplementary Fig. S13). Whether Ncb2 occupancy also impacts performance of other major TFs such as Ndt80, Mrr1, Upc2 and Ada2 contributing to MDR in *C. albicans*, remains to be explored^27^,^30^,^55^,^56^. In addition, we also analyzed the promoter regions (250 bp around the detected peaks for the genes that exhibited differential positional occupancy in AS and AR isolates) for the presence of other TFs motif, comprising transcriptional regulators of MDR such as Tac1, Ndt80, Mrr1, Ada2 and Upc2. In this context our preliminary analysis revealed no cis-elements in the promoter regions analyzed.

## Discussion

Continuous exposure to the antifungal drugs are recognized as environmental cues that triggers different mechanisms to develop drug tolerance and in the selection of the drug-resistant strains with the capacity to permanently withstand high thresholds of drug concentrations^57^. Therefore, understanding the molecular basis of the mechanisms that control the development of drug tolerance is of utmost importance^55^. In this context, we have earlier identified a novel mechanism of transcriptional regulation by a heterodimeric regulator Ncb2 which activate the transcription of *CDR1* in AR clinical isolate of *C. albicans*^43^. Ncb2 is a part of heterodimeric NC2 complex which also comprises NC2α. NC2 directly interacts with TBP and prevent its association to TFIIB and TFIIA to inhibit transcription^37^,^58^. Genome-wide expression analysis has revealed that NC2 is involved in transcriptional repression and to a lesser extent in transcriptional activation^36^,^59^. Mechanistic details how NC2 affects positive regulation of gene expression is largely unknown. In our previous report it was observed that Ncb2 occupied core promoter upstream region in AS isolate whereas it positions at the core promoter region in AR isolate of *CDR1* thereby leading to the formation of alternative pre-initiation complex assembly for transcription initiation. This study has further examined the genome-wide role of Ncb2 in *C. albicans*, particularly in the context of MDR. Our genome-wide ChIP-on-chip occupancy of Ncb2 between genetically matched pair of AS and AR isolates provides interesting insights into the novel regulation of transcription in response to drug stress in *C. albicans*.

Extensive analysis revealed that the transcription regulation by Ncb2 is not only restricted to *CDR1* but also several genes across the entire genome show moderate to high occupancy of the regulator. It is also apparent that most of the MDR genes, which are co-regulated along with *CDR1* by Tac1, are also enriched and regulated by Ncb2. The select validation of the enrichment of Ncb2 data points that the higher occupancy of the regulator does not necessarily result in the transcriptional activation as the expression of several genes was also repressed. Notwithstanding, the dual role of Ncb2, the genome-wide occupancy profiling reaffirmed its role as a global regulator of gene expression^46^,^60^.

A comparative analysis of the genome-wide binding profile of Ncb2 between AS and AR isolates disclosed that in response to drug stress, Ncb2 selectively escapes or binds to the core promoters/TATA-box regions. There were host of genes whose promoters showed positional shift of bound Ncb2 at different positions between AS and AR isolates. For example, while Ncb2 recruited at the core promoters/TATA-box regions in AR isolate, it exclusively positioned upstream of the core promoters/TATA-box regions in a set of genes of AS isolate. Interestingly, it has been observed earlier that the binding of NC2 (NC2α + Ncb2) complex to the DNA bound TBP results in the mobilization of TBP away from the TATA box or allows it to escape and return to the TATA binding mode probably by inducing a conformational change in the TBP-DNA complex^42^. Our earlier data where a close interaction between Ncb2, NC2α and TBP has been demonstrated supports such a mechanism^43^. It appears that Ncb2 not only binds and stabilizes TBP to maintain the initiation factor at the promoter regions, but it also mobilizes the TBP on the DNA, possibly together with NC2α. This mode of action of Ncb2 may explain its dual functionality^42^.

There were also a set of genes, which showed exclusive occupancy of Ncb2 on their promoters in AR isolate. Interestingly, most of such genes showed positive regulation of transcription. These results imply that an exclusive occupancy of Ncb2 in the AR isolate may exert a positive impact on gene expression by recruiting active pre-initiation complex at the promoters. In comparison, the genes whose promoter regions showed comparatively higher enrichment of Ncb2 occupancy in the AR isolate resulting in an activation or repression, points that the higher occupancy may not always lead to positive regulation of the transcription since it also depends on the promoter occupancies of other transcriptional activators^46^,^60^. The exclusive positional shift of Ncb2 at the core promoters/TATA-box regions in a set of genes in the AR isolate represent fine tuning of the regulation of gene expression to adapt the pathogen to drug stress. The members of such group comprised not only well-known MDR genes but also include several uncharacterized genes. The higher expression observed following validation of select genes suggest that these could represent novel genes who have important role to play in MDR of *C. albicans*.

The analysis of Ncb2 bound DNA regions showed that the frequency of Ncb2 motif is significantly higher as compared to the random sequences. Importantly, Ncb2 motif was found to be distributed around the detected peaks and constitute A-rich sequence (Fig. 6c). This is similar to the sequences that are found for the promoter regions of upstream positional shift in AS isolate (Fig. 7b). Interestingly, the presence of approximately repeated patterns or motifs signifies that these are regulated by the same gene regulatory mechanisms. Additionally, conservation of Ncb2 binding sequences in AS isolate for the genes that showed the core promoter upstream binding indicated that certain changes occurred in the genome of AR isolate that ultimately lead to the re-positioning of Ncb2 at the core promoters/TATA box regions. This possibly results in the regulated gene expression to adapt the pathogen to combat the effect of the drug.

In conclusion, we demonstrate a new mode of action of Ncb2 in *C. albicans* in response to drug stress by alternating its binding to the core promoters/TATA regions to either activate or repress transcription. Since, negative or positive regulation by Ncb2 does not correspond to the strength of the promoter occupancy; it may probably depend on the downstream promoter occupancies of other co-activators. Considering that MDR is a multifactorial manifestation of many regulatory mechanisms, this study provides an example that a global regulator may actively be involved in MDR acquisition in *C. albican*s.

## Materials and Methods

### Strains and growth

*C. albicans* azole resistant (AR) and azole sensitive (AS) isolates were grown in YEPD medium (1% yeast extract, 2% peptone, and 2% dextrose) in 30 °C incubator at 200 rpm for routine experimental purposes. Strains details are given in Supplementary Table S11.

### Primers

All the primers used in this study are listed in Supplementary Table S12.

#### ChIP-chip amplification, labeling, hybridization and data analysis

ChIP experiments were carried out as described earlier^43^. In brief, 15 ml of cultures in YEPD medium (AS and AR cells) were cross-linked with 1% formaldehyde for 20 min at RT. The cross-linking reaction was quenched with 125 mM glycine. Cells were washed twice with ice cold TBS (Tris Buffered Saline: 20 mM Tris-Cl, pH 7.5, containing 150 mM NaCl) and resuspended in Zymolyase buffer (50 mM Tris, pH 7.4, 10 mM MgCl_2_, 1 M sorbitol, and 30 mM DTT) for the formation of spheroplast. Spheroplasts were washed twice with cold TBS containing 1 M sorbitol, resuspended in ChIP lysis buffer (50 mM HEPES-KOH, pH 7.5, 140 mM NaCl, 1 mM EDTA pH 8.0, 1% Triton X-100, 0.1% sodium deoxycholate, and 1 mM PMSF). SDS was added at a final concentration of 0.5%. Cells were sonicated for 25 sec at amplitude of 6 for seven cycles. After sonication cell suspensions were centrifuged and termed soluble total chromatin (STC). Fifteen μl samples were run on 1% agarose gel to check the size of the fragmented chromatin (approximate size ~250 bp to ~500 bp). STC was diluted 5 times with ChIP lysis buffer and pre-cleared. After pre-clearing the samples pre-immune serum (negative control) and anti-Ncb2 antibody was added at a final concentration of 2–5 μg and incubated O/N^43^. Next morning 20 μl packed volume of protein-A agarose beads were added, bound immune complexes were precipitated and washed twice with ChIP lysis buffer, TSE 150 (50 mM Tris-Cl, pH 8.0, 150 mM NaCl, 1% Triton X-100, 0.1% SDS, 1 mM EDTA, pH 8.0, and 1 mM PMSF), TSE 500 (50 mM Tris-Cl, pH 8.0, 500 mM NaCl, 1% Triton X-100, 0.1% SDS, 1 mM EDTA, pH 8.0, and 1 mM PMSF), buffer III (10 mM Tris-Cl, pH 8.0, 1 mM EDTA, pH 8.0, 250 mM LiCl, 1% NP-40, and 1% sodium deoxycholate), and finally with TE (10 mM Tris-Cl, pH 8, and 1 mM EDTA, pH 8.0). After washing, immunoprecipitated complexes were eluted in buffer containing 50 mM Tris-Cl, pH 7.5, 10 mM EDTA, pH 8.0, and 1% SDS at 65 °C O/N. The immunoprecipitated complexes and input DNA were treated with proteinase-K. The precipitated DNA were either extracted by PCI (phenol:chloform:isoamylalcohol) followed by ethanol precipitation using glycogen (30 μg) as a DNA carrier or purified using Qiagen PCR purification kit. The input and immunoprecipitated DNA were dissolved in 100 μl and 20 μl TE for the validation of ChIP-on-chip data or eluted in 70 μl of TE buffer for samples that were used for ChIP-on-chip experiments. In PCR analysis, amplifications were carried out for 25 to 28 cycles.

A high-density oligonucleotide microarray with 5000 bp upstream (promoters) and 500 bp downstream regions (genes) of *C. albicans* (SC5314) was used. It covered 6204 orfs, representing 99.7% of the total genome comprising 180000 unique 60 mer probes *in situ* synthesized using “SurePrint” technology (Agilent) that was optimized for DNA-protein binding studies. To study the Ncb2 genome-wide association, ChIPed DNA purified by Qiagen PCR purification kit send to Genotypic technology Bengaluru for linear amplification by LMPCR (ligation mediated polymerase chain reaction) and size distribution of amplified fragment (~250–500 bp) as well as yields were determined. LMPCR was performed by using 200 ng grams of input and immunoprecipitated DNA was blunted at 12 °C for 20 min using T4 DNA polymerase as per the Mammalian ChIP-on-chip Protocol Version 10.1 of Agilent Technologies. The blunted DNA was cleaned by phenol:chloroform extraction and the precipitated DNA were re-suspended in 25 μl of water and the entire DNA was ligated to linkers overnight. The linker-ligated DNA was subjected to two rounds of LMPCR as per protocol and the product was precipitated and re-suspended in 25 μl of water.

Two μg of input, ChIPed or immunoprecipitated DNA were labeled using Agilent Genomic DNA labeling kit PLUS and Cy3 (for ChIPed or immunoprecipitated DNA) or Cy5 (for input DNA) dUTP from Agilent. DNA yield and incorporation of label (specific activity) was measured using Nano Drop spectrophotometer. Labeled DNA were hybridized at 65 °C for 24 hr to Agilent 60 mer oligonucleotides. Hybridized arrays were scanned using high throughput Agilent scanner with “SureScan” technology, and process through automated feature extraction using Agilent feature extraction software. After quantification and normalization of the signals (ChIPed-DNA or immunoprecipitated DNA/input DNA), the binding profiles were represented as normalized log ratios. Normalization of data and statistical analysis were performed using Agilent’s DNA analytics software (Agilent genomic workbench lite edition 6.5; ChIP-on-chip application, and Microsoft excel). Normalization methods used were median blanks subtraction and intra-array (intensity-dependent) LOWESS normalization. In blanks subtraction normalization for each channel (IP or input) of each replicate of each array, the median of the intensities of the blank spots is subtracted from each intensity on that replicate. As in all normalization steps, any negative signal intensities that result will cause the probe to be flagged as ‘excluded.’ Intra-array normalization attempts to correct for artifacts caused by nonlinear rates of dye incorporation, as well as inconsistencies in the relative fluorescence intensity between some red and green dyes. The LOWESS (locally weighted scatter plot smoothing) algorithm normalizes the channels within each array using a nonlinear polynomial fit to the data, and effectively normalizes by probes and by arrays. Whitehead per-array neighborhood model was used to detect the protein-DNA binding event in DNA Analytics software (DNA analytics detects robust peaks of probe signal corresponding to binding events). This method uses the distribution of all probes on each array to compute robust regions of increased probe signal (termed ‘peaks’). The algorithm does this by examining groups of probe triplets which are significantly enriched, yielding robust binding event detection. The Whitehead model samples every probe and its immediate upstream or downstream neighboring probe to identify a robust estimate of the location of bound protein. Three ChIPed DNA samples were processed for each strain. The data sets that support the results of this article are available in the GEO repository [ http://www.ncbi.nlm.nih.gov/geo/] with accession number GSE768558 for the tiling array data.

#### Isolation of RNA and RT PCR

Total RNA from AS and AR isolates were extracted by using Trizol reagent (Sigma). Briefly, cells from 15 ml of log phase cultures were centrifuged and washed twice with DEPC treated water. Three hundred mg of acid washed sterilized beads were added with 1 ml of Trizol reagent. Bead beating was done for 1 min, 3 times at 1 min interval on ice. After bead beating, 200 μl of chloroform was added, cell suspensions were mixed and centrifuged at 13 K for 10 min. Clear supernatants were taken out and 600 μl of isopropanol was added in each sample and stored at −20 °C O/N. Next day, samples were centrifuged at 13 K for 10 min at 4 °C and RNA pellet was washed with 75% ice cold ethanol. Samples were air dried and dissolved in 30 μl of DEPC treated water. Reverse transcription-PCR (RT-PCR) was performed as mentioned in the RevertAidTMH Minus kit (MBI, Fermentas). Briefly, a total of 10 μg RNA was treated with DNase for 30 min at 37 °C. Reactions were terminated by adding 1 μl of 25 mM EDTA, pH 8.0, and incubated further at 65 °C for 10 min. One μl of DNA free total RNA (1 μg) was primed with oligo(dT)18 and random hexamers for first strand c-DNA synthesis at 42 °C for 60 min. Reverse transcription reaction was terminated by heating at 70 °C for 5 min. Half a microlitre of c-DNA was used in PCR (25 μl reaction volume) primed with specific forward and reverse primers. Twenty-five to thirty cycles of amplifications were carried out to synthesize second strand unless otherwise stated. *ACT1* was used as an endogenous control. For every RT-PCR analysis, c-DNA was synthesized from RNA isolated from three different preparations. The PCR-amplified products were analyzed by agarose gel electrophoresis and quantified by densitometry.

#### Ncb2 consensus motif discovery

To find out the consensus binding site of Ncb2, the promoter sequences were analyzed with Regulatory sequence analysis tool (RSAT, http://rsat.sb-roscoff.fr) and Multiple EM for Motif Elucidation (MEME) program (http://meme-suite.org/tools/meme). For the analysis of Ncb2 consensus motif, 500 promoter sequences of the genes that showed highest Ncb2 binding normalized log ratio were selected. Three hundred bp sequence of the promoter regions were chosen around the detected peaks. The program was allowed to discover motif with oligo and position analysis with oligomer length above 6–7 bp and five motifs per algorithm searched on both strands. To find out the Ncb2 consensus sequence for the genes that showed core promoters/TATA-box upstream binding in AS isolate, 250 bp sequences were used around the detected binding peak. Furthermore, for the analysis of conserved sequences in the AR isolate in which Ncb2 binds at core promoters/TATA-box regions, 125 bp upstream and downstream sequences were taken from the ATG codon.

#### CRIP assay

The chromatin restriction digestion-coupled immunoprecipitation (CRIP) assay was performed as described earlier for the ChIP assay, with minor changes as described^43^. STC was fragmented by sonication to have a size of ~250 to ~500 bp. The Fragmented STC was subjected to restriction digestion (*PDX1* promoter with HinFI, *DFI1* with EcoRV, *ADAEC* with XhoI and *ECE1* with DpnI). Before digestion, chromatin was precipitated at room temperature by using 1 volume of isopropanol, and the pellet was recovered after centrifugation at 8,000 rpm for 10 min at room temperature and was washed twice with 70% alcohol at room temperature. The air dried pellet was solubilized in 1X universal Fast digest buffer, and 10 μl of respective restriction enzyme was added. Digestion was performed O/N at 37 °C. After completion of restriction digestion, chromatin was precipitated by absolute ethanol and dissolved in ChIP lysis buffer. ChIP experiment then was performed as described earlier.

## Additional Information

**Accession codes:** GSE768558.

**How to cite this article:** Shariq, M. *et al*. The global regulator Ncb2 escapes from the core promoter and impacts transcription in response to drug stress in *Candida albicans. Sci. Rep.* **7**, 46084; doi: 10.1038/srep46084 (2017).

**Publisher's note:** Springer Nature remains neutral with regard to jurisdictional claims in published maps and institutional affiliations.

## Supplementary Material

Supplementary Information
