# Supplementary material for: The global regulator Ncb2 escapes from the core promoter and impacts transcription in response to drug stress in Candida albicans
Source: Sci Rep. 2017 Apr 6;7:46084. doi: 10.1038/srep46084 (PMC5382705; doi:10.1038/srep46084)
Supplement: Supplementary Information [file srep46084-s1.pdf]

The global regulator Ncb2 escapes from the core promoter and impacts transcription in response to drug stress in *Candida albicans*

Mohd Shariq[1&2], Sanjiveeni Dhamgaye[3], Remya Nair[1&4], Neha Goyal[1],Vaibhav Jain[5], Arnab Mukhopadhyay[5], Alok K. Mondal[1], Gauranga Mukhopadhyay[2#] and Rajendra Prasad[6#]

[1]School of Life Sciences, Jawaharlal Nehru University, New Delhi-110067, INDIA.

[2]Special Centre for Molecular Medicine, Jawaharlal Nehru University, New Delhi-110067, INDIA.

[3]Department of Microbiology, Monash University, VIC, AUSTRALIA.

[4]Rajive Gandhi Institute of I.T and Biotechnology, Bharati Vidyapeeth University, Pune-411045, INDIA

[5]Molecular Aging Laboratory, National Institute of Immunology, Aruna Asaf Ali Marg, New Delhi-110067, INDIA.

[6]Amity Institute of Integrative Sciences and Health and Amity Institute of Biotechnology, Amity University, Haryana-122413, INDIA.

[#]For correspondence. E-mail: rp47jnu@gmail.com; gmukho1@gmail.com

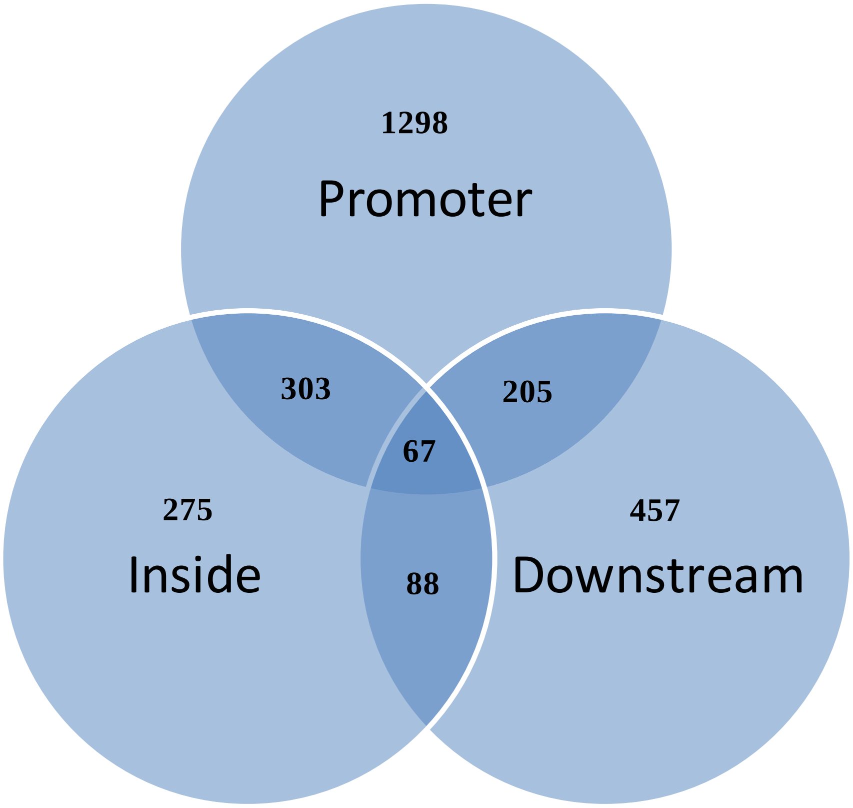

Supplementary Figure S1: Ncb2 binds at promoter, inside and downstream of the genes. Vein diagram showing the number of genes that are occupied by Ncb2 at promoter, inside and downstream of the genes in Gu4 (AS) isolate.

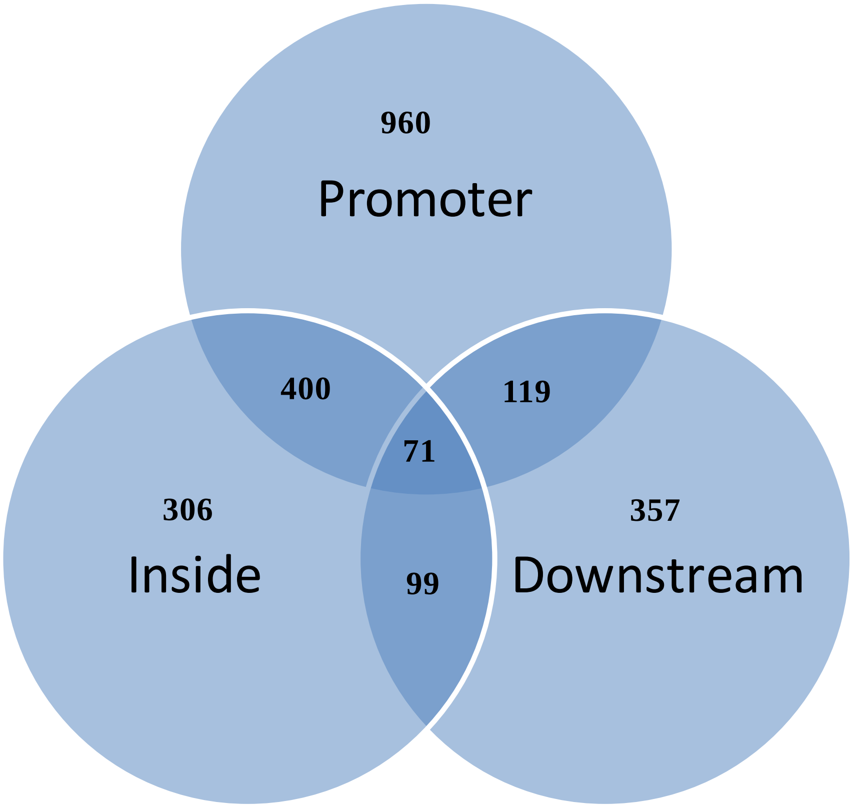

Supplementary Figure S2: Ncb2 binds at promoter, inside and downstream of the genes. Vein diagram showing the number of genes that are occupied by Ncb2 at promoter, inside and downstream of the genes in Gu5 (AR) isolate.

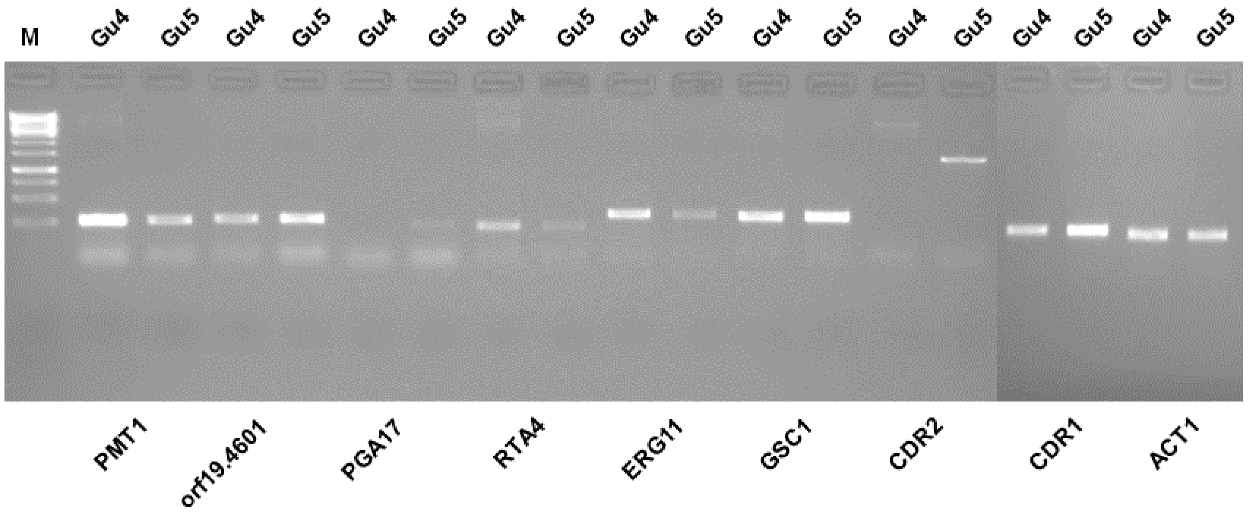

Supplementary Figure S3: Expression analysis of highly enriched genes of AR isolate as compared to AS isolate. Semi-quantitative end point RT-PCR was used to study the expression pattern of Gu5 highly enriched genes. *CDR1* and *CDR2* were used as positive controls. *ACT1* was used as control that equally expressed in both the isolates.

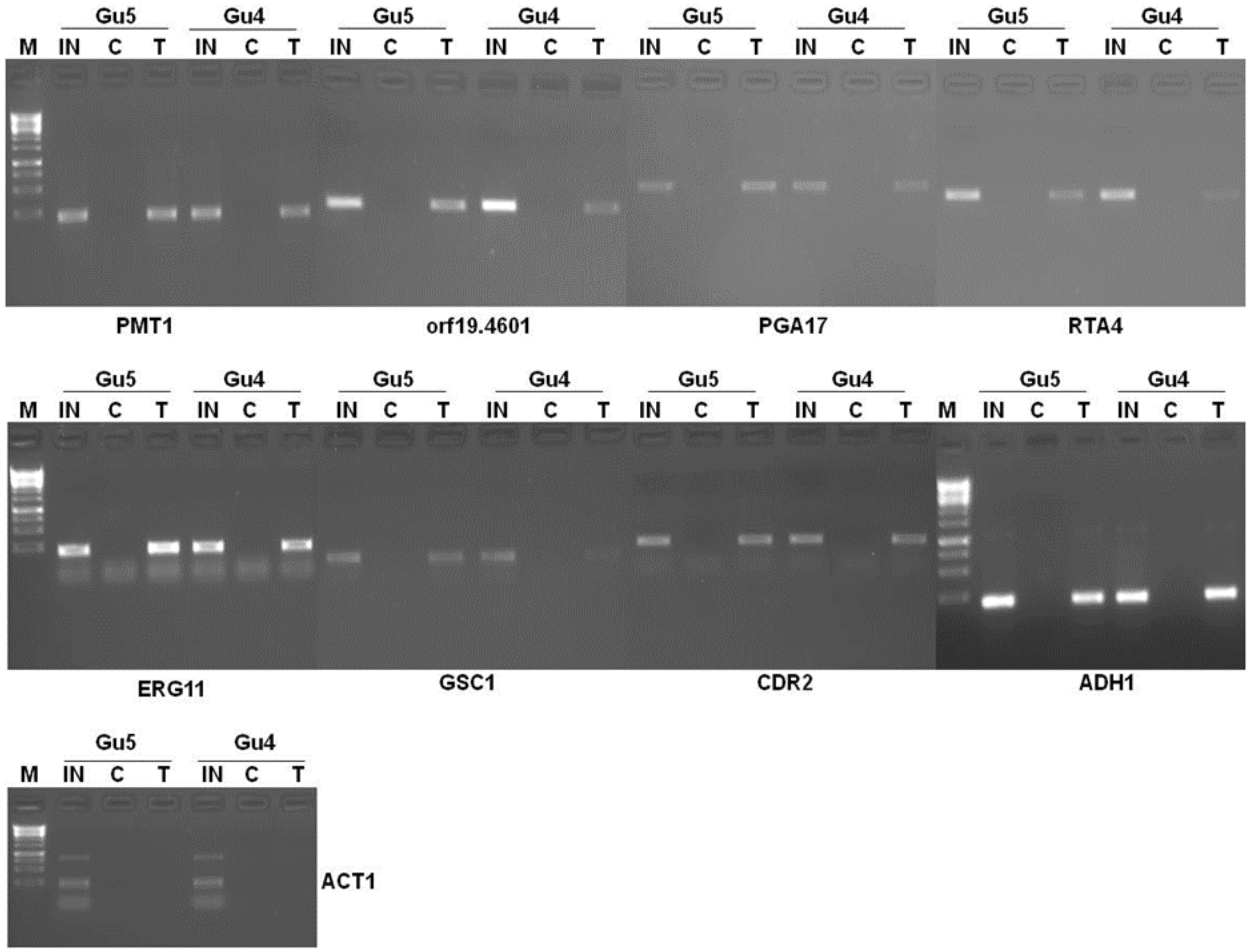

Supplementary Figure S4: Recruitment dynamics of AR highly enriched genes by Ncb2 as compared to AS isolate. Chromatin-immunoprecipitation (ChIP) showing Ncb2 enrichment in AR and AS isolates. Enrichment at *ADH1* and *ACT1* promoters were used as positive and negative controls for Ncb2 binding. IN, C, and T indicate input, control (ChIP using pre-immune serum), and test (ChIP using anti-Ncb2 antibody), respectively. M indicates molecular size marker (GeneRuler 1 kb DNA ladder from Thermo Fischer Scientific).

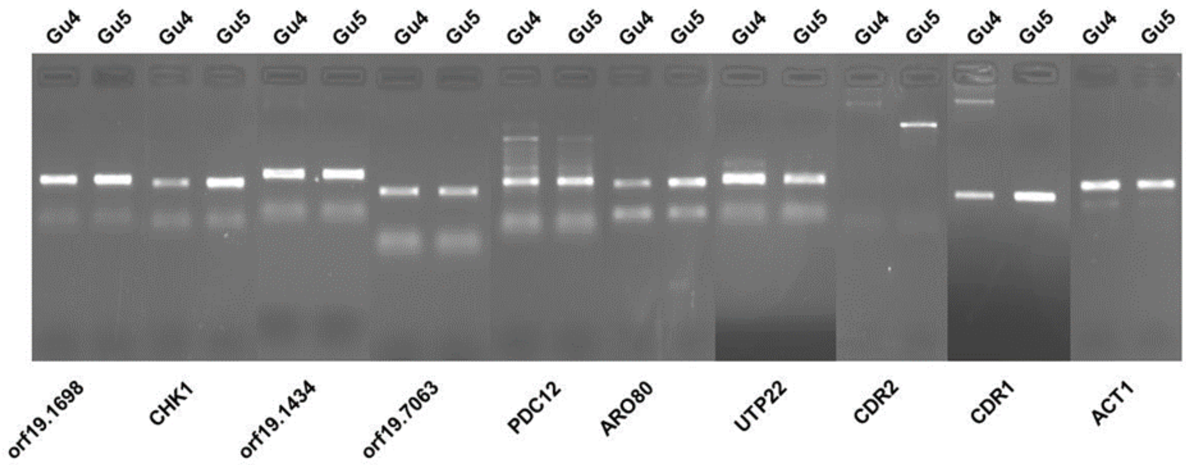

Supplementary Figure S5: Expression analysis of AR exclusive Ncb2 enriched genes. Expression of AR exclusive Ncb2 enriched genes was analyzed by semi-quantitative RT-PCR. Expression of *CDR1* and *CDR2* were used as positive controls. Expression of *ACT1* gene was used as endogenous control that equally expressed in both the isolates.

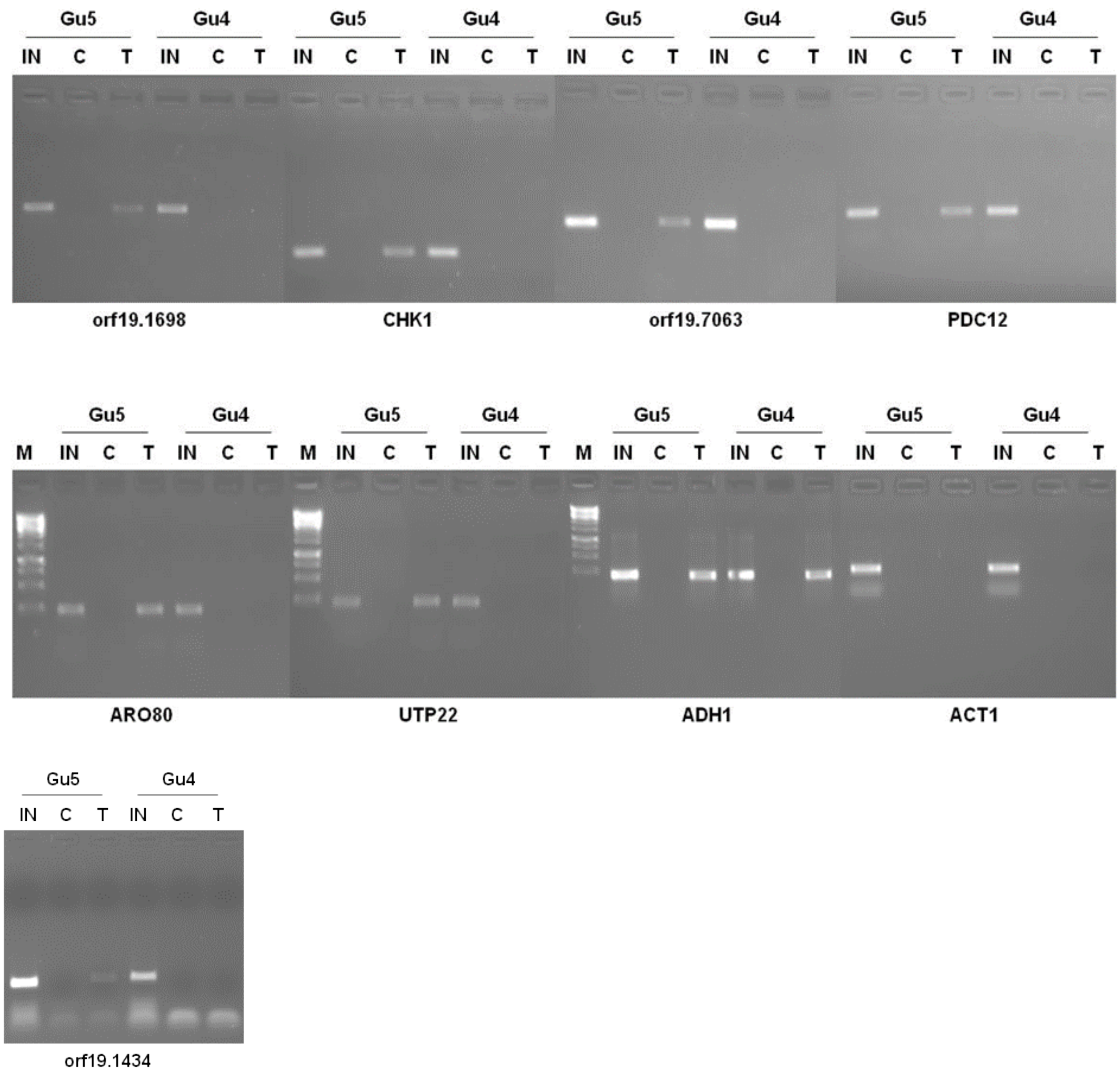

Supplementary Figure S6: Ncb2 exclusively occupied a set of genes in AR isolate. Chromatin-immunoprecipitation by using anti-Ncb2 antibody demonstrated Ncb2 exclusive occupancy in AR isolate. *ADH1* and *ACT1* were used as positive and negative controls for Ncb2 binding in both the isolates. IN, C, and T indicate input, control (ChIP using pre-immune serum) and test (ChIP using anti-Ncb2 antibody), respectively. M indicates molecular size marker (GeneRuler 1 kb DNA ladder from Thermo Fischer Scientific).

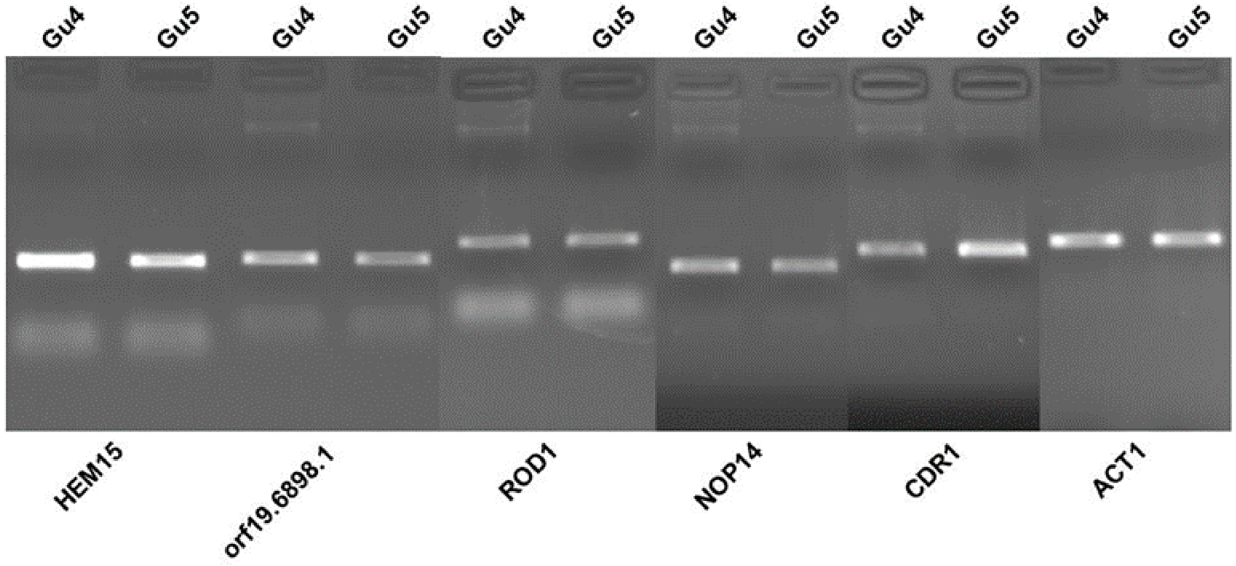

Supplementary Figure S7: Ncb2 exclusively occupied host of genes in AS isolate. Agarose gels demonstrating expression pattern of AS exclusive enriched genes. Semi-quantitative RT-PCR was used to study the difference in the expression pattern in both the isolates. *CDR1* and *ACT1* expression was used as positive and negative controls.

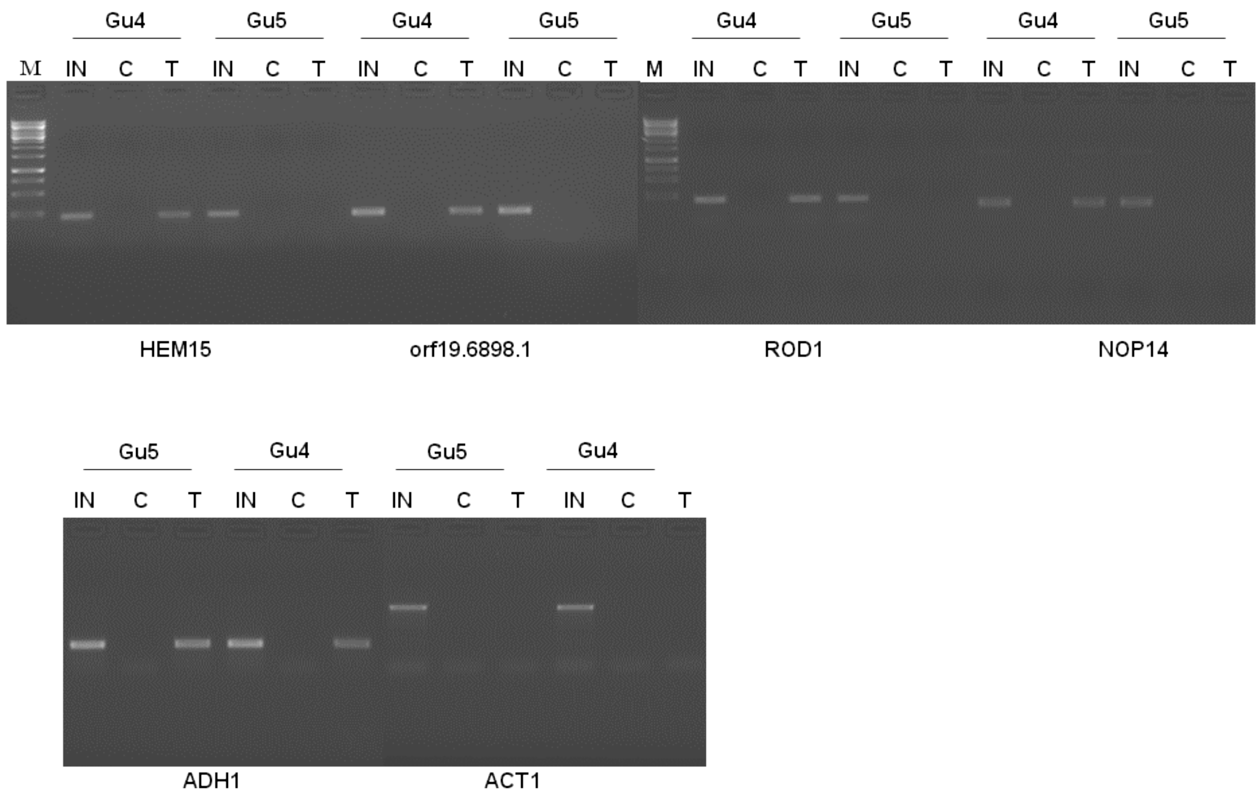

Supplementary Figure S8: Ncb2 enrichment profile of AS exclusive genes. ChIP was used to study the Ncb2 enrichment profile of AS exclusive genes. *ADH1* and *ACT1* were used as positive and negative controls for Ncb2 binding in both the isolates. IN, C, and T indicate input, control (ChIP using pre-immune serum), test (ChIP using anti-Ncb2 antibody), respectively. M indicates molecular size marker (GeneRuler 1 kb DNA ladder from Thermo Fischer Scientific).

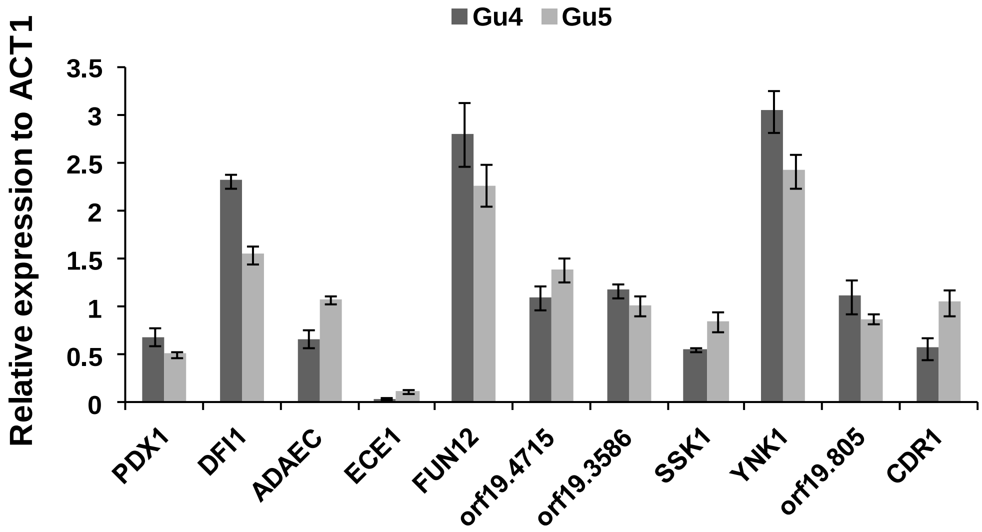

Supplementary Figure S9: Ncb2 occupancy at core promoter regions in AR isolate display upstream positional shift in AS isolate and showed modulated gene expression. Semi-quantitative RT-PCR result showing the expression profile of genes that showed positional shift in Ncb2 occupancy. *ACT1* gene expression was used to normalize the data. Bars represent the standard deviations observed for the replicate experiments. Expression of *CDR1* was used as a positive control for genes that over-expressed in AR isolate.

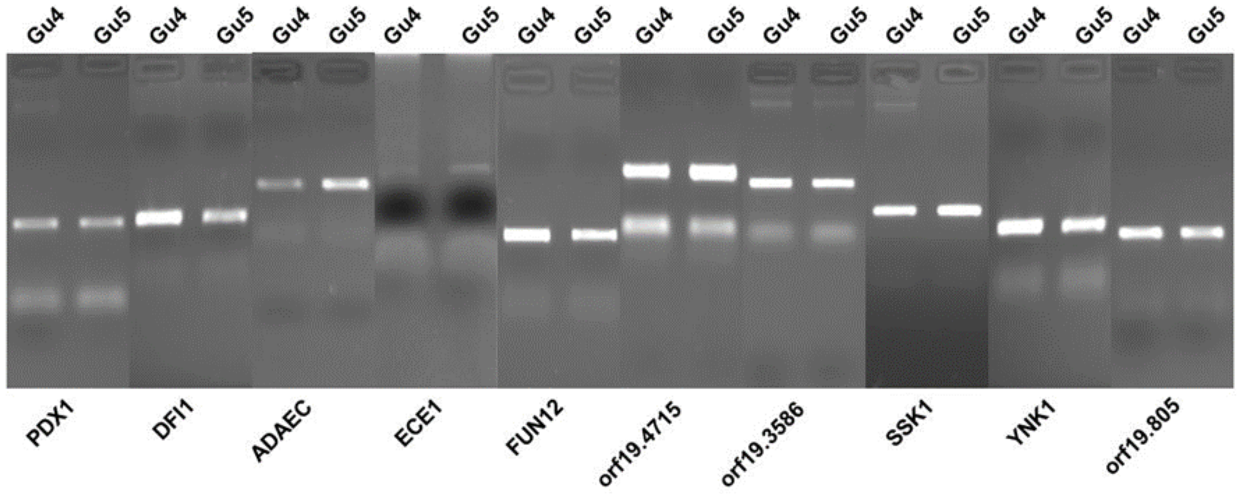

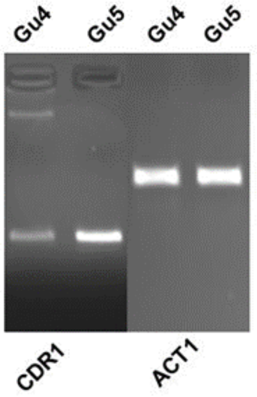

Supplementary Figure S10: Ncb2 occupied different positions at promoter regions of number of genes in AR and AS isolates. RT-PCR was used to study the difference in expression pattern in both the isolates. *CDR1* and *ACT1* expression was used as positive and negative controls.

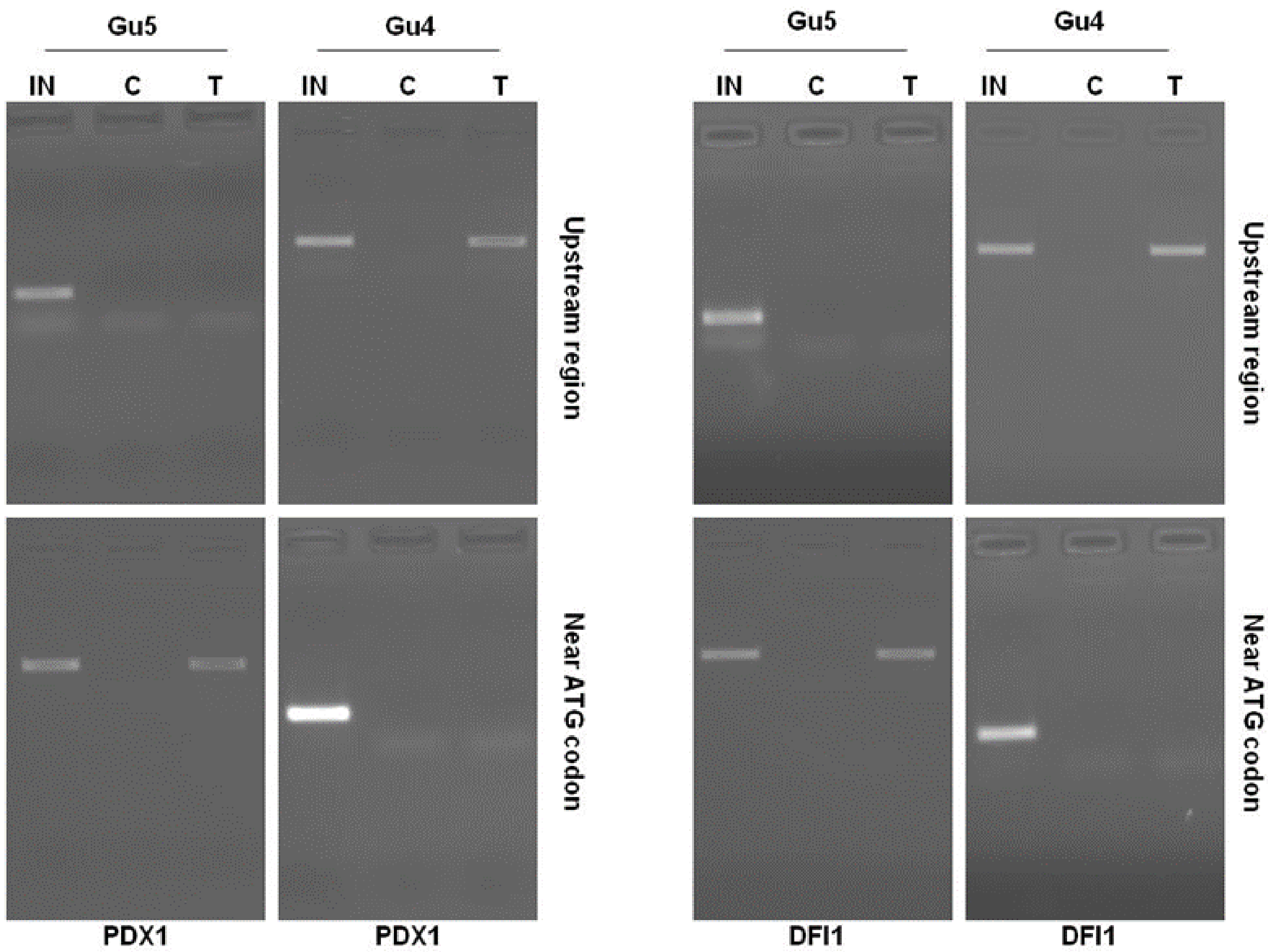

Supplementary Figure S11: Ncb2 occupied different positions at promoter regions of *PDX1* and *DFI1* in AS and AR isolates. Chromatin restriction digestion couple immunoprecipiation (CRIP) experiments depicting the recruitment of Ncb2 at the core promoter upstream region of *PDX1* and DFI1 genes in AS isolate whereas recruitment was found at core promoter region near ATG codon in AR isolate. IN, C, and T denote input DNA and immunoprecipitation found with control pre-immune serum and anti-Ncb2 antibody, respectively.

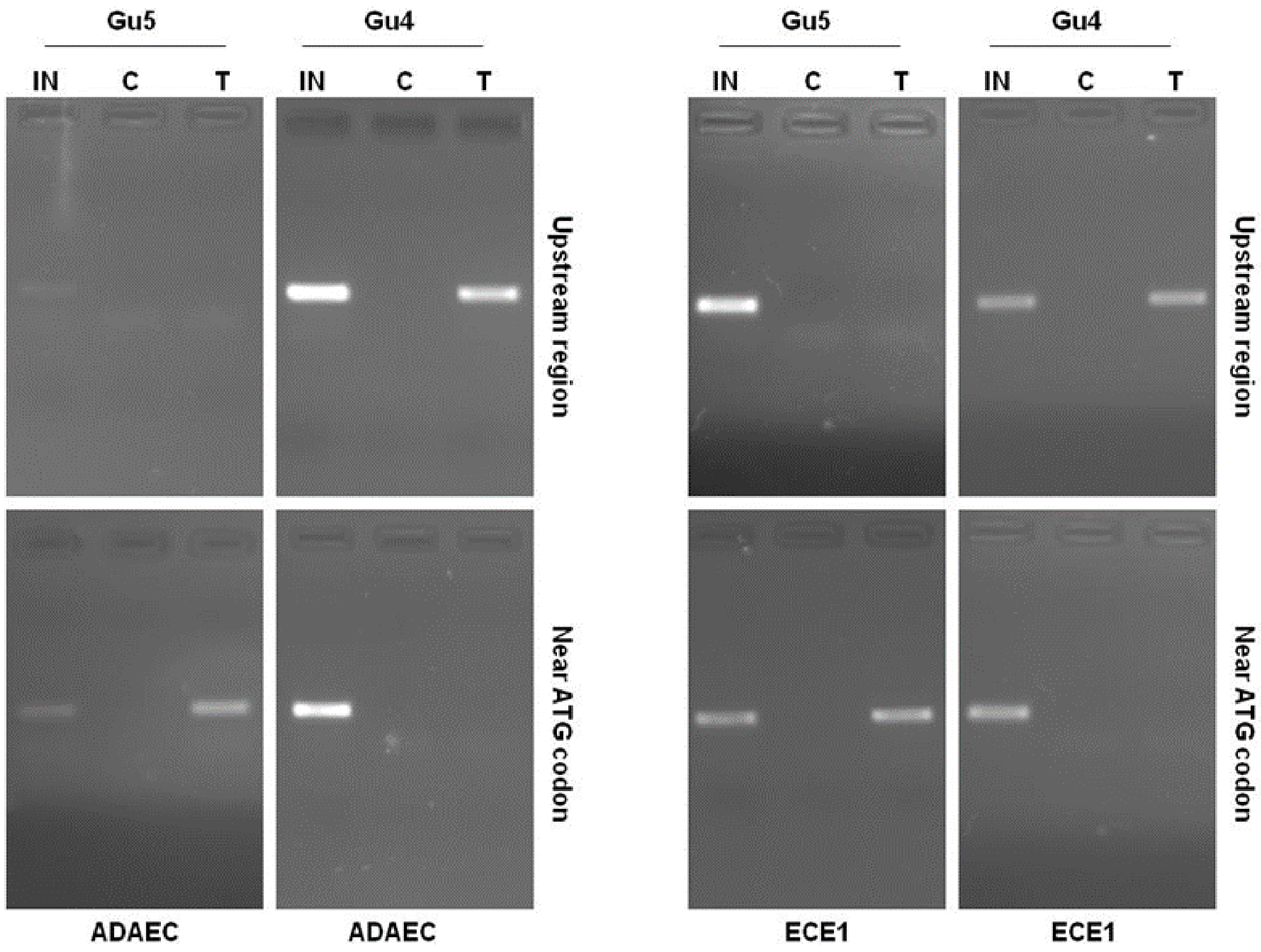

Supplementary Figure S12: Ncb2 occupies different positions at promoter regions of *ADAEC* and *ECE1* in AS and AR isolates. CRIP assay demonstrating the recruitment of Ncb2 at the core promoter upstream region of *ADAEC* and *ECE1* genes in AS isolate whereas its occupancy was found at core promoter region near ATG codon in AR isolate. IN, C, and T denote input DNA and immunoprecipitation found with control pre-immune serum and anti-Ncb2 antibody, respectively.

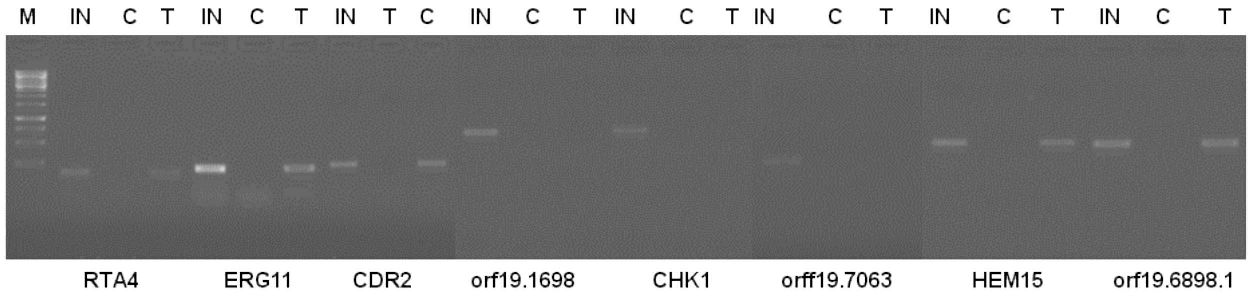

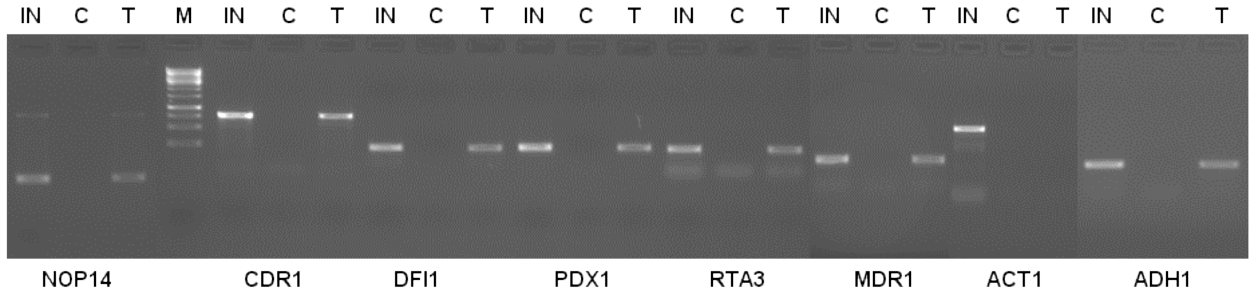

Supplementary Figure S13: Ncb2 enrichment profile of MDR genes in *Δtac1* strain. ChIP assay was used to study the Ncb2 recruitment dynamics at the promoters of MDR genes in *Δtac1* strain. *ADH1* and *ACT1* were used as positive and negative controls for Ncb2 binding. IN, C, and T indicate input, control (ChIP using pre-immune serum), test (ChIP using anti-Ncb2 antibody), respectively. M indicates molecular size marker (GeneRuler 1 kb DNA ladder from Thermo Fischer Scientific).

Supplementary Table S1: Functional Categories (Gu4 (AS) isolate, 1119 genes having *P*-value less than or equal to 0.01 and fold enrichment greater than or equal to 2.

(a) **Functional category**	**Gene name**
Related to DNA	EAF3, RAD54, ISW2, RAP1, ESA1, NHP6A, orf19.839, TEA1, PHO23, orf19.2903, SGS1, SIR2, orf19.6197, orf19.1052, HTA
Response to osmotic stress	RHR2, RCK2, orf19.4287, AQY1
Response to oxidative stress	SOD1, PRX1, TRR1, HSP60, orf19.3319, PBS2, TRX1, TAL1
Core stress protein	GRP2, NPR1, HGT6, UBI4, HAP41, AHP1
ATP biosynthesis process	YBN5, ATP3
Metabolism	MET16, orf19.3982, CHO1, FAS1, ACH1, FCA1, SAM2, SUR2, orf19.5565, orf19.1167, SAH1, orf19.7077, orf19.7522, STD1, PFK1, STP2, NPT1, AUR1, GUT2, PIKA, ACO1, GPD1, ILV2, FOX2, FBP1, PGK1, MNN4, FDH3, SPT23, INO2, CAR2, RGT1, UGA3, PFK2, GFA1, orf19.6306, orf19.6423, GCR3, MLS1, orf19.634, URA1, SRB1, URE2, LPD1, OLE1, MTS1, GPH1, ENO1, FBA1, GNA1, TDH3, GPM1, PDC11, ADH1, PDC11
Multidrug transport	FCR1, MDR1, CDR4, MDL2, FLU1
Transporter	NUP, GAP6, OPT1, NGT1, HGT19, orf19.7296, GIT1, orf19.3232, HGT2, ITR1, GAP5, PMC1, CRP1, NCE102, CCH1, OPT6, orf19.341, FTR1, PTR2, HGT1, HGT20, RTA2, PMA1, PDR16, HGT7, TPO3, orf19.93, HNM1, GEF2, CNT, FRP3
Virulence	UTP15, ZCF21, RVS161, EFG1, NOP14, PHR2, IFD6, YPT72, SET3, CKB2, NOC4, GIG1, RBT4, IPT1, NRG1, ICL1, SPA2, PTC6, orf19.6396, orf19.3228, LAG1, OCH1, TPS2, RVS167
Mitochondria associated	COX8, orf19.4966, orf19.6435, ATP7, orf19.3029, NDE1, ATP1, MRP2, COX4, TOM70, orf19.4190, orf19.3691, NAM2, orf19.5628, POR1
Morphogenetic role	SFL1, WOR2, orf19.5671, orf19.6783, CUP9, ECM25, orf19.2397, LAP41, orf19.676, IFF6, SLA2, CTA3, orf19.467, ROB1, WH11, ZCF5, SWE1, MYO1, ZCF7, STP4, ARC35, orf19.3335, FKH2, ADH5, POX1, WOR1, LSP1
Filamentous growth	RAS2, orf19.6748, CHA1, orf19.5576, RAD9, RNR1, IAH1, orf19.1617, FGR3, LIG4, KEM1, TUP1, FGR17, SHA3, HGT4, orf19.4246, orf19.5984, DPM1, RFG1, HEX3,

	orf19.4459, RAC1, TCC1, orf19.6874, orf19.1691
Carbon source utilization	CTN1, KIS2, CTF1
Amino acid metabolism	STP3, LYS4, HIS1, LYS12, LYS144, LYS1, LYS2, MET6, GLY1, GLC7
Protein folding/ heat shock	RBP1, orf19.6082, orf19.4346, MSI3, HSP70
Ergosterol metabolic process	ERG5, ERG26, ERG6, ERG11, HMO1, NCP1
Essential for cell viability	CDC37, PGA52, SEC14, orf19.4601, TKL1, SEC1, CUP1
Biofilm formation	orf19.4579, orf19.4617, CHT3, MKC1, TEC1, STT3, ALP1, DPP3, AMO2, PTK2, IFH1, QDR1
Signal transduction	RHO1, GSP1, CNB1, HSL1, YPD1, orf19.7149, orf19.5045, orf19.7239, orf19.1795.1, CMK1, orf19.7370, RCH1, GPR1
Cell wall	ROT2, RBT1, AMS1, ECM33, MNT1, NIK1, RHD3, CHT1, GPI7, SIM1, BMT4, PGA56, SCW11, PGA62, CHS4, BMT1, SLK19, SSR1, BGL2, RBE1, PGA59, PPZ1, RHB1, YWP1, KRE6
Adherence	WAR1, FCR3, ARA1, orf19.4062, SAP10, SUC1, ZCF31, UGA33, UTR2, CSH1, orf19.6824, ZCF39, AHR1, SAP9, MP65, CBP1
Cell cycle	CSE4, PCL5, TEM1, YOX1, CLB2, CLN3, orf19.6022, SWI4
Hyphal protein	RIM8, HYR1, DFG5, GAP4, SEC2, BRG1, orf19.633, SSN6, PGA7, MYO5, CCN1, CZF1, BNI4, YCK2, GAR1, VRG4, YTH1, MSS4, CHS7, RAD6, KIC1, CGR1, HGC1, PDI1, SSY1, XYL2
Protein translation	RPS10, SUI2, RPL4B, RPP1A, SUI1, SEC62, orf19.5953, RPL27A, RPS16A, orf19.997, HTS1, OST1, RPL6, RPS7A, RPL14, VPS36, RPP2B, RPS1, ASC1, PRT1, CYP1, RPL17B, RPL13, EGD2, TSR1
Proteasome activity	YUH2, SMT3, UBI3, UBA1
Mating	RAT1, SST2, CPH1
Heme/copper related	HEM3, HEM1, FLC1, FRE10, ORM1
Growth defects	MVB12
Apoptosis	MCA1
Trafficking	VPS27
Uncharacterized genes	orf19.264, orf19.6578, orf19.787, RPS20, SAM4, YOR1, HSM3, orf19.3003, orf19.4376, orf19.675, ROD1, RPS26A, SYS1, orf19.450, orf19.5502, orf19.6770, HTA2, orf19.1171, orf19.131.2, orf19.2468, orf19.4450.1, orf19.4783, orf19.5381, orf19.5989, orf19.6480, orf19.721, orf19.7499, RCT1, RPN3, orf19.1240, orf19.1486, orf19.1735, orf19.2670, orf19.2691, orf19.2826, orf19.5074, orf19.5293, RAX1, HAP3, IHD2, orf19.6080, CDC21, COF1, DAL52,

	orf19.3134, orf19.6357, PHO87, COX19, DPP1, GCY1, orf19.2701, orf19.3264.1, orf19.36.1, orf19.3665, orf19.3697, orf19.3806, orf19.5711, orf19.670.2, orf19.6929, YCF1, MAL31, orf19.3265.1, orf19.4395, orf19.4805, orf19.5019, orf19.5728, orf19.6219, PST3, RPS21, orf19.1274, orf19.6266, orf19.6636, orf19.808, RLI1, MSN4, orf19.1203.1, CTF5, CWH8, orf19.1397, orf19.3059.1, orf19.4883, orf19.6003, ATP1, orf19.3644, orf19.6118, orf19.7548, orf19.149, orf19.4607, PCL7, RPL43A, SMD3, ACF2, CAN3, orf19.252, orf19.3007, orf19.3610, orf19.4779, orf19.5834, orf19.6268, orf19.7196, orf19.7437, HGT16, orf19.1239, orf19.3648, orf19.4643, orf19.792, IML2, orf19.1389, orf19.5334, orf19.5943.1, orf19.6919, RPL8B, ERG25, orf19.1862, orf19.2509.1, orf19.5289, orf19.5626, orf19.5925, orf19.6793, RPL37B, orf19.2246, orf19.323, orf19.364, orf19.3751, orf19.3915, orf19.5625, YPT31, orf19.1219, orf19.1433, orf19.2024, orf19.3869, orf19.5525, RPP0, MRPL33, orf19.1381, orf19.1956, orf19.5917.3, BUD31, BUL1, orf19.1085, orf19.2933, orf19.3406, orf19.5003, RPS18, ASG1, orf19.2757, orf19.2905, orf19.2973, orf19.4900, orf19.4942, RXT3, SNX4, ERG20, MCR1, orf19.2459, orf19.3782.2, orf19.3968, orf19.4210, orf19.5866, orf19.684, orf19.1267, orf19.4699, PEX14, CDC47, MAF1, MDM34, orf19.3076, orf19.4525, orf19.6816, CPY1, HHF22, IFD3, orf19.4121, orf19.577, orf19.7502, PUF3, RNR22, SPE3, ARG3, orf19.3460, orf19.4148, orf19.4727, orf19.53, orf19.5860, PHM5, RPS17B, orf19.2296, orf19.2529.1, orf19.5620, orf19.7151, PGA63, GLT1, IHD1, orf19.1736, orf19.5229, orf19.6681, PUT1, MDH1-1, orf19.3916, orf19.5322, orf19.3984, orf19.411, orf19.5169, orf19.6110, orf19.6644, PGI1, ADK1, orf19.1535, orf19.3325, orf19.4445, orf19.6275, orf19.6873.1, orf19.7288, orf19.7310, orf19.813, orf19.1767, orf19.1972, orf19.2367, orf19.3456, orf19.4595, orf19.4672, orf19.4903, SOD6, orf19.1440.2, orf19.4820, TEF4, orf19.1137, orf19.35, orf19.5201.1, SWD2, AXL2, CDC34, MDH1-3, orf19.448, orf19.4914.1, orf19.6264.4, KGD2, LSC1, orf19.3572.3, orf19.4263, orf19.4882, orf19.1212, orf19.938, RPL28, FGR37, orf19.2766, orf19.5035, TFA1, orf19.2943.5, orf19.3782, orf19.5205, orf19.6980, LEU42, orf19.3439, orf19.6013, PRO3, RPL39, orf19.2789, orf19.6288, orf19.6982, orf19.7304, RSN1, MLP1, orf19.6554, SBP1, DPP2, orf19.3437, CLG1, DLD1, orf19.3684, orf19.3689, orf19.649, TIF5, orf19.699, orf19.7279.1, ALD5, MEU1, orf19.3897, orf19.5504, PGA60, RPL5, orf19.242.2, orf19.3229, orf19.6458.1, RPL2, RPS15,

	CDR11, MNN11, MOH1, orf19.1356, orf19.3314, orf19.3983, XKS1, ACS1, orf19.263.1, ECM21, orf19.1204, orf19.6484, orf19.6491, orf19.6973, TPS3, orf19.3216, orf19.6983, orf19.1943, RPL21A, CAM1, orf19.338, PTH2, TOS1, orf19.6246, orf19.6260, orf1395, orf19.2328, orf19.3690.2, orf19.4835, orf19.4921.1, PRP8, orf19.6882.1, APE3, HHT2, ISN1, orf19.1910, orf19.5161, orf19.5980, PPS1, orf19.4850, RPS28B, orf19.1776, orf19.6740, HTB1, orf19.1229, orf19.6592, RPS23A, FUN31, orf19.1504, orf19.3148, orf19.6348, orf19.6810, STF2, orf19.1409.1, orf19.3455, orf19.1060, EMC9, orf19.2178.1, orf19.3215, orf19.4264, orf19.7341, DAK2, orf19.3156, orf19.3626.1, RPL40B, SPO75, orf19.2888, orf19.4659, orf19.6310, GDB1, IPL1, orf19.2934, orf19.399, orf19.7357, orf19.7380, HXK2, orf19.1796, orf19.215, orf19.4959, orf19.4748, orf19.4906, orf19.599, orf19.6984, orf19.1991, orf19.3973, SEC12, orf19.1387, TLO8, XUT1, CPA2, GSY1, orf19.5828, orf19.7199, orf19.1191, orf19.6456, orf19.3482, OSM1, orf19.4070, orf19.1075, orf19.3728, orf19.1675, orf19.5465, orf19.1082, orf19.2575, orf19.1344, TAR1, RPL30, ILV5, orf19.2825, PST1, PGA61, orf19.6604, orf19.6688, orf19.7337, orf19.7269, LEU1, orf19.7497, orf19.5799, orf19.6979, TLO34, orf19.7108, orf19.6030, INN1, orf19.6027, orf19.51, PGM2, orf19.1765, orf19.3505, STB3, RGS2, RPS12, orf19.3428, RPT2, HBR2, PEX17, orf19.1785, SPE2, orf19.5754, orf19.6192, orf19.3475, orf19.4629, orf19.6114, orf19.6115

(a) Functional categorizations of genes were done according to description given at Candida genome database.

Supplementary Table S2: Functional Categories (Gu5 (AR) isolate, 960 genes having *P*-value less than or equal to 0.01 and fold enrichment greater than or equal to 2.

(a) **Functional category**	**Gene name**
Related to DNA	EAF6, ZCF17, NHP6A, ESA1, ISW2, orf19.6197, SIR2, PHO23, RAP1, SGS1, HTA1, orf19.2903, GIN1, orf19.1052
Response to osmotic stress	orf19.4287, RCK2, AQY1
Response to oxidative stress	EBP1, GZF3, TRR1, SOD1, orf19.3319, PRX1, HSP60, TRX1, PBS2, TAL1
Core stress protein	DRS1, GRP2, HGT6, UBI4, HAP41, AHP1
Metabolism	GUT2, NPT1, orf19.556, orf19.3982, SPT23, ILV2, URA1, PIKA, FBP1, CAR2, STD1, ACH1, orf19.6423, INO2, GPD1, PGK1, GPM1, CHO1, PFK1, MTS1, orf19.6306, MLS1, FAS1, RGT1, SRB1, ACO1, GFA1, GPH1, URE2, GNA1, LPD1, OLE1, FOX2, orf19.634, TDH3, ENO1, FBA1, ADH1
Multidrug transport	CDR4, MDR1, MDL2
Transporter	GIT1, orf19.341, FTR1, HGT2, CRP1, HGT7, NUP, PMC1, NCE102, PHM7, HGT1, FRP3, GAP6, orf19.323, CNT, GEF2, HGT20, PMA1, FCY21, OPT6, PTR2, orf19.93, TPO3, PDR16, RTA2, VPH1, HNM1
Virulence	FAS2, ADE2, NOP1, SPA2, PTC1, ZCF21, HSX11, NOC4, IFD6, EFG1, GIG1, orf19.6396, DUR1,2, PTC6, SET3, RVS167, ICL1, PHR2, TPS2, RBT4, orf19.3228, LAG1
Mitochondria associated	orf19.6435, COX4, MRP2, TOM70, POR1, NDE1, orf19.4190, orf19.3691, orf19.5628
Morphogenetic role	CUP9, WOR2, ZCF5, orf19.2761, orf19.33, orf19.6783, ARC35, orf19.3335, WH11, ECM25, ADH5, STP4, orf19.676, MYO1, FKH2, LSP1, SWE1, POX1
Filamentous growth	orf19.5576, orf19.557, PHHB, MAL2, IAH1, SSU1, KEM1, RAS2, orf19.5984, RTA4, RPB7, TUP1, orf19.55, RAC1, FGR3, TCC1, CHA1, orf19.6874, orf19.4246, HEX3, SHA3, FGR17, HGT4, orf19.4459, orf19.1691, RFG1, FCR1
Carbon source utilization	CTF1, CTN1, KIS2
Amino acid metabolism	LYS144, LYS1, MET6, LYS2, GLY1, GLC7
Protein folding/ heat shock	orf19.6082, HSP104, orf19.4346, SSC1, HSP70
Ergosterol metabolic process	ERG26, ERG251, ERG5, ERG9, ERG11, NCP1
Essential for cell	SEC1, TKL1, SEC14, HMO1, orf19.4601, CUP1

viability	
Biofilm formation	MKC1,RIX7, PLB3, CHT3, orf19.3603, STT3, orf19.4617, TEC1, OBPA, QDR1, ALP1, AMO2, PTK2, IFH1, DPP3, orf19.2447
Signal transduction	orf19.7370, orf19.1795.1, RAS1, HSL1, BOI2, YPD1, GPR1, CMK1, RCH1
Cell wall	SUR7, ECM331, ECM3, SCW11, MNT1, NIK1, ECM33, BMT4, RBE1, SIM1, GPI7, PGA56, CHS4, KRE6, BMT1, BGL2, PGA62, PPZ1, RHB1, YWP1, PGA59
Adherence	orf19.4062, ZCF31, ARA1, SAP10, EAP1, AHR1, MP65, orf19.3434, WAR1, CSH1, orf19.5975, ZCF39, orf19.6824, SAP9, CBP1
Cell cycle	CLN3, PCL5, YOX1, CLB2, TEM1, SWI4, orf19.6022
Hyphal protein	RIM101, VPS51, XYL2, RIM8, MYO5, CZF1, orf19.633, MSS4, YCK2, PTP3, GAP4, CCN1, BNI4, CGR1, RAD6, PGA7, YTH1, CHS7, SSY1, KIC1, HGC1, PDI1
Protein translation	RPP2A, SUI2, MAK21, orf19.5953, SEC62, HTS1, orf19.997, OST1, RPP2B, RPS7A, RPS16A, EGD2, PRT1, RPL27A, ASC1, RPL2, CYP1, RPL17B, RPS1, TSR1
Proteasome activity	SMT3, UBA1, UBI3
Mating	RAT1, CPH1, SST2
Heme/copper related	FLC1, MAC1, HEM3, FRP1, FRE10, ORM1
Growth defects	orf19.2760, MVB12, FMO1
Apoptosis	MCA1
Trafficking	VPS27
Uncharacterized genes	orf19.5468, orf19.6852.1, PGA23, TIF, orf19.2757, orf19.3456, orf19.6578, orf19.6929, URA2, orf19.1943, orf19.1994, orf19.5282, orf19.6984, orf19.3606, orf19.4883, GLT1, orf19.6264.4, orf19.6770, orf19.7596, PPH21, HTB1, IHD2, orf19.1728, orf19.3134, orf19.3782.2, orf19.4672, orf19.53, orf19.3043, orf19.4942, orf19.5381, orf19.6498, orf19.697, CIS2, orf19.1219, orf19.2973, orf19.3325, orf19.3572.3, orf19.3806, orf19.6110, orf19.6194, orf19.7288, POL32, REG1, orf19.1535, orf19.2959.1, orf19.2844, orf19.3332, orf19.3525, orf19.6268, PHA2, orf19.1106, orf19.1769, orf19.2204.2, orf19.2529.1, orf19.3897, orf19.5289, orf19.6802, TPO2, MDM34, orf19.1267, orf19.3610, SOD6, orf19.1240, orf19.1444, orf19.6982, PCK1, orf19.2778, orf19.5161, orf19.5293, orf19.36.1, orf19.3984, orf19.6003, orf19.6681, orf19.951, YTP31, APT1, PCT1, ARO80, CDR11, orf19.3076, orf19.411, orf19.6219, orf19.6458.1, ACS1, IHD1, orf19.2246, TLO8, orf19.2826, ECM21, orf19.1440.2, orf19.35, orf19.6869, TPS3, DPS1-1, orf19.1972, orf19.3983, orf19.6227, RPC31, MEU1, orf19.2037, orf19.2887, orf19.5541, orf19.6986, RPS21, orf19.6816, LSC1,

	orf19.2468, orf19.1736, orf19.4900, orf19.6288, orf19.6973, RME1, HGT16, IFD3, orf19.2024, orf19.4450.1, orf19.5943.1, orf19.6920, COX19, HHF22, HAP3, LYS142, MDH1-3, orf19.1303, orf19.4148, orf19.1821, orf19.2943.5, orf19.6266, orf19.7304, TLO7, CDC34, orf19.3455, orf19.649, orf19.1757, orf19.2905, orf19.6260, ARH2, orf19.1397, orf19.1946, DAO2, FGR37, orf19.6872, orf19.6873.1, CDC21, FAA4, orf19.14433, orf19.5504, orf19.6310, RSN1, STF2, CAN3, KGD2, orf19.4376, orf19.5980, orf19.6941, RPS17B, orf19.2888, orf19.6852, SBP1, orf19.1085, orf19.2825, orf19.263.1, orf19.4488, RNR22, orf19.1191, orf19.4263, orf19.6601, SNX4, MOH1, orf19.5322, orf19.6013, PTH2, RCL1, RXT3, orf19.5625, orf19.5917.3, orf19.6591, DPP2, orf19.3007, orf19.5860, orf19.2459, orf19.4607, PGA63, SPE3, SWD2, ACF2, orf19.3418, orf19.3968, orf19.5205, ADK1, CLG1, orf19.3644, PST1, orf19.5019, orf19.6919, orf19.2575, ATP1, MAF1, orf19.7279.1, NIP7, orf19.192, orf19.2725, orf19.364, MDH1-1, orf19.5828, RPL28, CDC47, CPA1, FUN31, LEU42, orf19.2178.1, orf19.2691, orf19.4121, orf19.4659, PHO89, APE3, orf19.1060, orf19.675, orf19.1330, orf19.3437, ISN1, orf19.4081, orf19.6592, orf19.6275, orf19.6980, orf19.938, PRO3, RCT1, TOS1, CPY1, CWH8, orf19.4850, orf19.4959, orf19.6348, orf19.6810, orf19.7199, orf19.3689, orf19.813, orf19.1203.1, orf19.2766, orf19.3406, orf19.6644, orf19.1356, HTA2, MNN11, SPO75, orf19.4445, orf19.2509.1, DLD1, orf19.2789, orf19.338, PGA61, orf19.6491, orf19.7380, XKS1, orf19.1767, orf19.4921.1, RPL37B, orf19.1212, orf19.3869, HHT2, CAM1, orf19.1210, orf19.4070, MLP1, orf19.3302, orf19.3720, orf19.3439, orf19.7357, LEU1, orf19.5229, RPN3, TAR1, ILV5, orf19.1796, EMC9, orf19.149, orf19.4903, GDB1, orf19.1387, PPS1, orf19.4264, orf19.450, RPS23A, orf19.3059.1, RPL21A, GSY1, orf19.3973, orf19.4699, orf19.448, orf19.6246, orf19.2328, RPL30, TEF4, orf19.7341, STB3, orf19.7497, orf19.7108, orf19.1776, orf19.6554, orf19.1910, orf19.399, orf19.1409.1, orf19.7269, orf19.2934, orf19.1991, orf19.4914.1, RPS12, orf19.1229, orf19.5626, OSM1, orf19.215, orf19.7337, orf19.1504, orf19.6027, orf19.6983, orf19.6030, orf19.51, orf19.3690.2, orf19.3156, orf19.6688, MSN4, RPL40B, orf19.3148, orf19.242.2, orf19.4906, orf19.3626.1, PUT1, PGM2, INN1, orf19.1675, orf19.6114, orf19.1665, HXK2, orf19.1075, HBR2, SEC12, orf19.1344, orf19.3728, RGS2, orf19.6604, orf19.1486, orf19.5799, orf19.3505, TLO34, orf19.1785, orf19.6793, RPT2, orf19.3428, SPE2, PEX17, orf19.6192, orf19.3475

(a) Functional categorizations of genes were done according to description given at Candida genome database.

Supplementary Table S3: Gu5 (AR) highly enriched genes.

**List of Gu5 (AR) highly enriched Ncb2 occupied genes in comparison to Gu4 (AS) isolate**			
	**Gu5 (AR)**	**Gu4 (AS)**	
**Gene name**	(a) **Average Normalized log ratio**	(a) **Average Normalized log ratio**	(b) **Binding distance from ATG**
ERG11	2.5403316	1.8172877	596
orf19.4601	2.406287	1.565620133	377
PFK1	2.3337066	1.608176067	228
GSC1	2.154144	1.553946067	1939
IFU5	2.088118767	1.4309268	201
orf19.1785	2.051259233	1.264197467	265
orf19.6227	2.022317767	1.1673856	71
ZCF17	1.9695309	0.868730467	109
orf19.1678	1.966247067	1.14794815	149
KRE62	1.838073167	1.2315313	1115
CDR2	1.809403333	1.18642065	18 inside
UBA4	1.7577269	1.263400433	117
orf19.134	1.725593833	1.010412833	321
FCY21	1.700761233	1.071825367	907
WSC2	1.6834452	1.052062	1233
orf19.133	1.675051433	0.79801453	301
PMT1	1.6672137	1.140241467	38 inside
RTA4	1.461414267	0.921887	200
RAS1	1.447387767	0.995811387	572
PGA17	1.4219034	0.908954	21
ATF1	1.413	1.016	238
KGD1	1.61	0.593	13
THS1	1.61	0.785221687	26 inside
HEM2	1.28873478	0.56588914	190
orf19.4358	1.720290367	1.085413053	25
PGA54	1.216384833	0.818610033	742
ILV3	1.39	0.873	212
orf19.29	1.7	0.926	103
ERG12	1.967672733	1.193333733	125

(a) Average normalized log ratios were calculated from three individual experiments for both the isolates for the same probe that has minimum Min P[X bar] value.

(b) Binding distance from the ATG codon were calculated for both the isolates for the probe that has minimum Min P[X bar] value.

Supplementary Table S4: go term mapper result of Gu5 (AR) highly enriched genes.

**go TermMapperResult, Gu5 (AR) highly enriched Ncb2 occupied genes as compared to Gu4 (AS)**				
GOID	GO term	Frequency	Gene(s)	Organism
8150	biological_process	10 out of 29 genes, 34.5%	CR_03870W_A C6_01260W_A C6_01250W_A PGA54 C1_06820W_A C3_01660W_A KRE62 IFU5 PGA17 C2_09980W_A	Candida albicans
42221	response to chemical	7 out of 29 genes, 24.1%	CDR2 GSC1 ERG11 UBA4 FCY21 RAS1 PMT1	Candida albicans
6950	response to stress	7 out of 29 genes, 24.1%	CDR2 WSC2 RTA4 UBA4 C2_06530W_A RAS1 PMT1	Candida albicans
50789	regulation of biological process	7 out of 29 genes, 24.1%	WSC2 ERG11 UBA4 ZCF17 C2_06530W_A RAS1 PMT1	Candida albicans
6810	transport	5 out of 29 genes, 17.2%	CDR2 FCY21 RAS1 PFK1 PMT1	Candida albicans
42493	response to drug	5 out of 29 genes, 17.2%	CDR2 GSC1 ERG11 FCY21 PMT1	Candida albicans
30447	filamentous growth	5 out of 29 genes, 17.2%	RTA4 UBA4 ZCF17 RAS1 PMT1	Candida albicans
5975	carbohydrate metabolic process	3 out of 29 genes, 10.3%	GSC1 PFK1 PMT1	Candida albicans
16070	RNA metabolic process	3 out of 29 genes, 10.3%	C4_01910W_A THS1 UBA4	Candida albicans
71555	cell wall organization	3 out of 29 genes, 10.3%	GSC1 WSC2 PMT1	Candida albicans

7049	cell cycle	3 out of 29 genes, 10.3%	GSC1 C2_06530W_A RAS1	Candida albicans
6464	cellular protein modification process	3 out of 29 genes, 10.3%	UBA4 RAS1 PMT1	Candida albicans
9405	pathogenesis	3 out of 29 genes, 10.3%	GSC1 RAS1 PMT1	Candida albicans
6629	lipid metabolic process	2 out of 29 genes, 6.9%	ERG12 ERG11	Candida albicans
48468	cell development	2 out of 29 genes, 6.9%	GSC1 RAS1	Candida albicans
6091	generation of precursor metabolites and energy	2 out of 29 genes, 6.9%	KGD1 PFK1	Candida albicans
7155	cell adhesion	2 out of 29 genes, 6.9%	RAS1 PMT1	Candida albicans
7165	signal transduction	2 out of 29 genes, 6.9%	WSC2 RAS1	Candida albicans
70783	growth of unicellular organism as a thread of attached cells	2 out of 29 genes, 6.9%	UBA4 ZCF17	Candida albicans
44419	interspecies interaction between organisms	2 out of 29 genes, 6.9%	RAS1 PMT1	Candida albicans
42710	biofilm formation	2 out of 29 genes, 6.9%	RAS1 PMT1	Candida albicans
3016	protein catabolic	1 out of	PMT1	Candida

3	process	29 genes, 3.4%		albicans
45333	cellular respiration	1 out of 29 genes, 3.4%	KGD1	Candida albicans
6412	translation	1 out of 29 genes, 3.4%	THS1	Candida albicans
7114	cell budding	1 out of 29 genes, 3.4%	UBA4	Candida albicans
746	conjugation	1 out of 29 genes, 3.4%	RAS1	Candida albicans
19725	cellular homeostasis	1 out of 29 genes, 3.4%	RAS1	Candida albicans
6259	DNA metabolic process	1 out of 29 genes, 3.4%	C2_06530W_A	Candida albicans

Supplementary Table S5: Gu5 (AR) exclusive Ncb2 enriched genes.

**List of exclusively enriched genes by Ncb2 in Gu5 (AR) isolate in comparison to Gu4 (AS) isolate**			
	**Gu5 (AR)**	**Gu4 (AS)**	
**Gene name**	(a) **Average Normalized log ratio**	(a) **Average Normalized log ratio**	(b) **Binding distance from ATG**
orf19.2447	2.73975	0.581582	167
VPH1	2.429961767	1.408860933	635
orf19.647.3	1.92117	-0.144183353	45
orf19.5755	0.801777	0.485633	514
ARO80	1.907055233	1.142629667	91
orf19.7368	1.72789321	1.124253057	23 inside
orf19.55	1.59865	0.72662007	231
CHK1	1.567881333	0.632590653	41
orf19.697	2.080804	1.293032	80
orf19.6269	1.473571	0.871628	426
orf19.1434	1.425471367	0.644392483	457
orf19.3831	1.387469433	0.686635617	22 inside
ERG9	1.385815633	0.77629702	153
orf19.1210	1.378125133	0.541353147	505
PDC12	1.359306433	0.607948887	41 inside
orf19.6450	1.351879033	0.421393433	195
orf19.3928	1.23080101	0.556374467	456
IFG3	1.228283113	0.463126647	234
orf19.7063	1.170113967	0.442096873	21 inside
orf19.2204.2	1.512956	0.370853	132
orf19.1698	1.785564	0.683986	34
UTP22	1.915113	0.8305	178
orf19.446	1.753149	1.065053	132
orf19.5614	0.8610178	0.4068365	256
RPT4	1.075707	0.596076	271

(a) Average normalized log ratios were calculated from three individual experiments for both the isolates for the same probe that has minimum Min P[X bar] value.

(b) Binding distance from the ATG codon were calculated for both the isolates for the probe that has minimum Min P[X bar] value.

Supplementary Table S6: Gu4 (AS) exclusive Ncb2 enriched genes.

**List of exclusively enriched genes by Ncb2 in Gu4 (AS) isolate in comparison to Gu5 (AR) isolate**			
	**Gu4 (AS)**	**Gu5 (AR)**	
**Gene name**	(a) **Average Normalized log ratio**	(a) **Average Normalized log ratio**	(b) **Binding distance from ATG**
CCH1	0.983153023	0.300124897	331
IRA2	1.639814533	0.054976827	526
orf19.4952.1	1.516461033	1.128245233	1564
HEM15	1.504723833	1.2397567	277
orf19.6612	1.453718967	1.366941533	83
orf19.3245	1.4503695	1.10378081	209
IMP4	1.428412767	0.63147621	132
CUP2	1.4080799	0.879491167	198
orf19.5978	1.394279433	1.331865367	166
BMT6	1.3644446	0.729861833	480
orf19.931	1.346426	0.47139041	254
orf19.6898.1	1.3334728	0.398003323	188
orf19.3945	1.315156333	0.361079793	725
orf19.4171	1.306198867	0.914555583	114
DAL52	1.2987264	0.515418133	2721
NOP14	1.282214333	0.223750813	128
orf19.7038	1.281835067	1.221603	100
ROD1	1.280826133	-0.376623623	603
RPL23A	1.2691766	0.08126291	193
FET99	1.2664683	0.593677592	363
PEX13	1.2516545	0.848308393	371
orf19.5813	1.192700233	0.706587967	318
orf19.1400	1.173968133	-0.286979685	329
orf19.3210	1.171776933	0.255880657	3311
orf19.5621	1.146601967	0.544347716	175
orf19.1730	1.128435967	0.81525501	439
ZRT2	1.115823633	0.073367887	284
SFL1	1.079966433	0.38968545	1423
orf19.4430	1.056431567	0.770089437	97
orf19.1995	1.055986597	0.327129157	1107
orf19.3259	1.0470086	0.50939651	13
ADE5,7	1.041259733	0.53525337	225

SMC1	1.003730267	0.533098603	314
orf19.7204	0.988519133	0.512946257	518
orf19.5578	0.981063033	0.885769437	4
orf19.6690	0.977286267	0.25549119	483
orf19.345	0.968672747	0.339446857	220
GTT13	0.962323703	0.342528543	355
SNO1	0.958289017	0.47321936	192
orf19.921	0.906420547	0.365187773	1779
orf19.6908	0.904686	0.583622933	99
orf19.7566	0.895838213	0.35216541	1005
ZCF24	0.889595073	0.317875867	22
orf19.5710	0.880716723	0.295208873	21
orf19.5495	0.86310521	0.206502697	1444
orf19.1604	0.8472649	0.463881573	903
orf19.5278	0.84277651	0.301326002	183
orf19.2068	0.8361888	0.279427153	85
orf19.3007.2	0.831320073	0.179930957	156
SRV2	0.80206538	0.089862447	94
CSH3	0.801655083	0.329591843	370
SEC18	0.796702683	-0.176690507	228
orf19.7301	0.782918467	0.230879977	151
orf19.5728	0.774599733	0.111090899	824
IFC1	0.768844107	-0.258317477	875
orf19.5556	0.7685386	0.303757373	199
orf19.1150	0.758642027	0.421678107	608
ADO1	0.752095217	-0.269706793	201
CDC50	0.712656813	0.5063771	902
orf19.4474	0.690433343	0.67272961	225
MNN7	0.664824617	0.578777543	805
CHT1	0.633780333	0.52262525	354
orf19.35.1	0.5926688	0.374599403	128
HGT5	0.39523206	0.294516647	229
APL2	0.318511113	-0.450047252	282

(a) Average normalized log ratios were calculated from three individual experiments for both the isolates for the same probe that has minimum Min P[X bar] value.

(b) Binding distance from the ATG codon were calculated for both the isolates for the probe that has minimum Min P[X bar] value.

Supplementary Table S7: go term mapper result of Gu4 (AS) exclusive Ncb2 enriched genes.

**go TermMapperResult, Gu4 (AS) exclusively enriched Ncb2 genes as compared to Gu5 (AR)**				
GOID	GO term	Frequency	Gene(s)	Organism
8150	biological_process	17 out of 65 genes, 26.2%	GTT13 ROD1 C1_03150C_A C5_04010C_A C2_06320W_A CR_01090W_A C2_00590W_A CR_09050C_A C2_09560C_A C7_01160C_A C5_00580W_A CR_09510C_A C4_00710W_A C2_06610C_A C6_02990W_A C6_02820W_A C7_03580C_A	Candida albicans
42221	response to chemical	14 out of 65 genes, 21.5%	C6_03330C_A ADO1 APL2 CUP2 CCH1 C3_03470W_A NOP14 C1_03990W_A SFL1 C2_02920W_A C5_04610W_A HMS1 SRV2 IRA2	Candida albicans
6810	transport	14 out of 65 genes, 21.5%	C6_03460W_A APL2 CR_09920W_A DAL52 CCH1 C7_00790W_A SEC18 PEX13 CSH3 ZRT2 CR_00990W_A HGT5 CDC50 OPT2	Candida albicans
50789	regulation of biological process	14 out of 65 genes, 21.5%	APL2 CUP2 CCH1 C2_09460C_A SFL1 SNO1 CSH3 ZRT2 C5_04610W_A HMS1 SRV2 ZCF24 C1_11690W_A IRA2	Candida albicans
6950	response to stress	9 out of 65 genes, 13.8%	CUP2 CCH1 C3_03470W_A CSH3 HMS1 SRV2 C3_01210C_A SMC1 IRA2	Candida albicans
42493	response to drug	7 out of 65 genes, 10.8%	C6_03330C_A ADO1 APL2 CCH1 NOP14 SFL1 SRV2	Candida albicans
30447	filamentous growth	7 out of 65 genes, 10.8%	CCH1 SFL1 CHT1 CSH3 HMS1 SRV2 ADE5,7	Candida albicans
6996	organelle organization	6 out of 65 genes, 9.2%	C6_03460W_A SEC18 PEX13 SRV2 C3_01210C_A SMC1	Candida albicans
16192	vesicle-mediated transport	5 out of 65 genes, 7.7%	APL2 C7_00790W_A SEC18 CSH3 CDC50	Candida albicans
16070	RNA metabolic	4 out of 65	IMP4 NOP14 C5_04610W_A ZCF24	Candida albicans

	process	genes, 6.2%		
9405	pathogenesis	4 out of 65 genes, 6.2%	CSH3 HMS1 SRV2 ADE5,7	Candida albicans
42254	ribosome biogenesis	3 out of 65 genes, 4.6%	C6_03460W_A IMP4 NOP14	Candida albicans
5975	carbohydrate metabolic process	3 out of 65 genes, 4.6%	CHT1 MNN24 MNN26	Candida albicans
30163	protein catabolic process	2 out of 65 genes, 3.1%	C1_03990W_A C1_07390W_A	Candida albicans
6629	lipid metabolic process	2 out of 65 genes, 3.1%	BMT6 PEX13	Candida albicans
6457	protein folding	2 out of 65 genes, 3.1%	CSH3 C1_13260W_A	Candida albicans
7165	signal transduction	2 out of 65 genes, 3.1%	HMS1 SRV2	Candida albicans
6464	cellular protein modification process	2 out of 65 genes, 3.1%	MNN24 MNN26	Candida albicans
19725	cellular homeostasis	2 out of 65 genes, 3.1%	CCH1 ADE5,7	Candida albicans
6259	DNA metabolic process	2 out of 65 genes, 3.1%	C3_01210C_A SMC1	Candida albicans
7010	cytoskeleton organization	1 out of 65 genes, 1.5%	SRV2	Candida albicans

30448	hyphal growth	1 out of 65 genes, 1.5%	CCH1	Candida albicans
6412	translation	1 out of 65 genes, 1.5%	RPL23A	Candida albicans
6766	vitamin metabolic process	1 out of 65 genes, 1.5%	SNO1	Candida albicans
71555	cell wall organization	1 out of 65 genes, 1.5%	CCH1	Candida albicans
7049	cell cycle	1 out of 65 genes, 1.5%	SMC1	Candida albicans
746	conjugation	1 out of 65 genes, 1.5%	C5_04610W_A	Candida albicans

Supplementary Table S8: Ncb2 occupied Tac1 regulon genes in Gu4 (AS) and Gu5 (AR) isolates.

**Systematic name**	CGD/NRC name	**GO terminology**	Ncb2 enrichment (Gu4 isolate)	**Ncb2 enrichment (Gu5 isolate)**
**orf19.6000**	CDR1	**Transporter activity**	Yes	**Yes**
**orf19.6869**	AST2	**Peptidase activity**	Yes	**Yes**
**orf19.6627**			Yes	**Yes**
**orf19.1267**	CAJ1	**Chaperone regulator activity**	Yes	**Yes**
**orf19.1444**	ENT2	**Cytoskeletal adapter activity**	Yes	**Yes**
**orf19.5958**	CDR2	**Transporter activity**	Yes	**Yes**
**orf19.3455**	LPE10	**Magnesium ion transporter activity**	Yes	**Yes**
**orf19.86**	GPX2	**Glutathione peroxidase activity**	Yes	**Yes**
**orf19.3406**			Yes	**Yes**
**orf19.7166**			Yes	**Yes**
**orf19.7042**			Yes	**Yes**
**orf19.1027**	PDR16	**Phosphatidylinositol transporter activity**	Yes	**Yes**
**orf19.3395**			Yes	**Yes**
**orf19.5877**	ATF1	**Alcohol O-acetyltransferase activity**	Yes	**Yes**
**orf19.23**	RTA3	**Phospholipid translocating ATPase activity**	Yes	**Yes**
**orf19.6627**			Yes	**Yes**
**orf19.4898**			Yes	**Yes**
**orf19.691**	GPD2	**GAPDH (NAD+) activity**	Yes	**Yes**
**orf19.1665**	MNT1	**Alpha-1,2 mannosyl transferase activity**	Yes	**Yes**
**orf19.7306**			Yes	**Yes**
**orf19.7319**	SUC1	**Transcription factor activity**	Yes	**Yes**
**orf19.577**			Yes	**Yes**
**orf19.5525**			Yes	**Yes**
**orf19.4476**	IFD6	**Aryl-alcohol dehydrogenase activity**	Yes	**Yes**
**orf19.2568**	IFU5		Yes	**Yes**
**orf19.93**			Yes	**Yes**
**orf19.1718**	ZCF8		Yes	**Yes**
**orf19.951**			Yes	**Yes**
**orf19.5257**	LCB4	**D-Erythro sphingosine kinase activity**	Yes	**Yes**

Supplementary Table S9: Genes that showed different binding position in Gu5 (AR) and Gu4 (AS) isolate.

**List of genes that showed Ncb2 positional shift in Gu4 (AS) and Gu5 (AR) isolates**					
	**Gu4 (AS)**	**Gu4 (AS)**	**Gu4 (AS)**	**Gu5 (AR)**	**Gu5 (AR)**
**Gene name**	(a) **Average Normalized Log Ratio**	**Ncb2 binding sequence**	(b) **Binding away from ATG**	**(a) Average Normalized Log ratio**	**Binding away from ATG**
		**5'-3'**			
CDR1	1.039082113	TGTAAACAAAATGA	974	0.973334	17 inside
orf19.511	0.793376833	ATCAAAGAAAATAA	698	1.8041444	26
orf19.7368	0.947486857	GGCAAACAAAAAAA	695	1.72789321	23 inside
orf19.805	0.829607287	GTCGAAAAAACAAA	264	1.582572233	15 inside
orf19.4715	1.662923	TTGGAAAAAAAAAA	250	1.491398267	9 inside
CIT1	1.4257834	AGCAAAAAAAAAAA	902	1.4347295	1 inside
orf19.3586	0.816724	AGGAAAAAAAAAAA	298	1.411088367	27 inside
PDX1	0.981159467	ACCAAAAAAAAAAA	584	1.332938867	14 inside
EFT2	0.96145865	GCGAAAAAAAAAGA	255	1.3225465	16 inside
orf19.1658	1.6760965	ATTGAAAAAAAAAA	462	1.31541102	72
SAH1	1.660517367	GCGAAAAAAAAAGA	255	1.2921771	15 inside
LCB4	1.2044978	ACCGAAAAAAATAA	181	1.220981467	16 inside
PGA45	0.731314973	CCCCAACAGAAAAA	840	1.204955233	25 inside
YNK1	0.809839367	GGCGAAAAAAAAAA	163	1.20390502	2 inside
RVS162	1.068713333	AGAAAAGAGAAAAA	734	1.0680691	194
ADAEC	1.1283737	GGTAAAAATAATAA	1015	1.098893953	63
CRZ2	0.864543547	AATGAAGATAATGA	2780	1.073599767	10 inside
GIT3	0.6476187	ATCAAAAAAAGAAA	993	1.064751167	213
ZCF8	0.881884223	GTAGAAGAAAATGA	1503	0.9998216	183
DFI1	1.0462758	AGCAAAAAAAAAAA	769	0.962118633	139
FUN12	1.1158536	AATGAAAAAAAAAA	227	0.946692167	53 inside
orf19.7459	0.938143577	GCTAAAGAAAAAAA	1467	0.946555167	7 inside

orf19.541	0.879241617	TACAAAAAAAGTAA	844	0.9316987	4 inside
MPT5	0.7149703	ACCAAAAAAAAAAA	2440	0.93144038	190
ECE1	1.230053033	CGCCAAGAAAAAAA	408	0.902568393	168
SSK1	1.3566357	ACCAAAAAAAAAAA	139	0.893320667	42 inside
NDH51	0.857818833	GGCGAAAAAAAAAA	1153	0.86407079	17 inside
orf19.4446	1.504356333	AGTGAAAAAAATAA	711	0.972157963	171
RTA3	1.5623553	GATAAAGAAAAAAA	338	0.782773513	7 inside
SUN41	0.63632814	GGACAAAAAAAAAA	2069	1.393946867	32 inside
orf19.5728	0.774599733	CCCCAACAATATAA	824	0.741363757	16 inside
orf19.1676	1.071001667	TAGGAAGAAAGAGA	480	0.65048851	0
CTA8	1.0908715	AGAGAAAAAAAAAA	849	0.645203683	22 inside
HEM1	1.5570958	CGGAAAAAAAAAAA	419	0.526869647	31 inside
NAB3	1.230225767	TGAAAAAAAAAAAA	183	0.630449597	28 inside

(a) Average normalized log ratios were calculated from three individual experiments for both the isolates for the same probe that has minimum Min P[X bar] value.

(b) Binding distance from the ATG codon were calculated for both the isolates for the probe that has minimum Min P[X bar] value.

Supplementary Table S10: go term mapper result for the genes that showed different positional occupancy in Gu4 and Gu5 isolates.

**go TermMapperResult, Genes that showed different positional occupancy in Gu4 and Gu5 isolates**					
GOID	GO term	Frequency	Gene(s)	Organism	
42710	biofilm formation	7 out of 35 genes, 20%	ZCF8 NDH51 C3_06700C_A SUN41 PDX1 CRZ2 ECE1	Candida albicans	
30447	filamentous growth	6 out of 35 genes, 17.1%	ZCF8 NDH51 C3_06700C_A SUN41 PDX1 SSK1	Candida albicans	
6950	response to stress	5 out of 35 genes, 14.3%	NDH51 CTA8 CDR1 SSK1 CRZ2	Candida albicans	
42221	response to chemical	4 out of 35 genes, 11.4%	CDR1 SSK1 CRZ2 EFT2	Candida albicans	
50789	regulation of biological process	4 out of 35 genes, 11.4%	ZCF8 CTA8 SSK1 CRZ2	Candida albicans	
8150	biological_process	4 out of 35 genes, 11.4%	C3_01890C_A C2_04160W_A PGA45 RVS162	Candida albicans	
6412	translation	2 out of 35 genes, 5.7%	FUN12 EFT2	Candida albicans	
6810	transport	2 out of 35 genes, 5.7%	GIT3 CDR1	Candida albicans	
71555	cell wall organization	2 out of 35 genes, 5.7%	SUN41 SSK1	Candida albicans	
42493	response to drug	2 out of 35 genes, 5.7%	CDR1 EFT2	Candida albicans	
44419	interspecies interaction between organisms	2 out of 35 genes, 5.7%	SUN41 SSK1	Candida albicans	
9405	pathogenesis	2 out of 35 genes, 5.7%	SUN41 SSK1	Candida albicans	
45333	cellular respiration	1 out of 35 genes, 2.9%	NDH51	Candida albicans	
6091	generation of precursor metabolites and energy	1 out of 35 genes, 2.9%	NDH51	Candida albicans	
7155	cell adhesion	1 out of 35 genes, 2.9%	DFI1	Candida albicans	
7165	signal transduction	1 out of 35 genes, 2.9%	SSK1	Candida albicans	

Supplementary Table S11: List of the strains used in the study.

Strains	Descriptions	Reference
Gu4	Fluconazole susceptible isolate	Franz, R., M., et al., 1999
Gu5	Fluconazole resistant isolate	Franz, R., M., et al., 1999
DSY2906	*tac1*Δ::*hisG*/*tac1*Δ::*hisG*	Coste, A., T., et al., 2004

Supplementary Table S12: Sequences of the primers used in the study.

Gene name	Primer sequence
CDR1 rt	FOR: AAGAGAACCATTACCAGG<br><br>REV: AGGAATCGACGGATCAC
GSC1 rt	FOR: TGCTTCGTCAAGATGGGCTGCT<br><br>REV: CACCCAATGGCATGACGGC
ERG11 rt	FOR: GCTATTGTTGAAACTGTCATTG<br><br>REV: GAAGCAGAAGTATGTTGACCA
RTA4 rt	FOR: CCATTGCTGCCACTTATGTTCCAG<br><br>REV: GGTGGCGGTGGCGGTGTTAA
CDR2 rt	FOR: CATGGTCAAGCCATTTTGTG<br><br>REV: ATCCATTCTGCTGGATTTGC
PGA17 rt	FOR: AAGAGACATGGCCTTCAGAGA<br><br>REV: ATAGTAATGACAGCACTGCTGGA
Orf19.4601 rt	FOR: ATAGGAGATATCATGGATGCAGA<br><br>REV: TCTGTCCTCGTGCATATCATTA
PMT1 rt	FOR: AGAATATGACATTCAAGAGAAATCAT<br><br>REV: AAGCATCTACATCAGCAACTTGT
ARO80 rt	FOR: CAAGATAATTCAAGTGGTAATGCA<br><br>REV: TCCATTTGTCCAGTATTAATTGC

PDC12 rt	FOR: GTAGCTGCCAAGGAAGTTGA<br><br>REV: GACTATCCTTAGCATTAAACAACAGC
Orf19.7063 rt	FOR: ATTTGCCCAACTTTGATGCA<br><br>REV: ATTCTCAAACTCTCTCACAATGATAGT
Orf19.1434 rt	FOR: ATGCCGGATATTACAGATTTGC<br><br>REV: CAGCTTCGAGTAATTTCCGTT
CHK1 rt	FOR: ATATTATTAGCAGAAGACAATCTTTTGA<br><br>REV: ACTTTCCTTTTTTTGTGTGTGAA
Orf19.1698 rt	FOR: AGAGGCAATACCCTAACTCATTC<br><br>REV: GATCGTATTCTTCAAGTTCAATAAATC
UTP22 rt	FOR: CAGGATTATGACTTCACTATTAAGGTC<br><br>REV: AATACCACCAATGACGTTTTCA
NOP14 rt	FOR: ACATATCACCAAATATAATGCCAC<br><br>REV: TAGTGCTGCCTTCTTTTCCTT
ROD1 rt	FOR: AATGCATTCAAGAACTGTATCAAGT<br><br>REV: GTTCGATCAGCAGCACTATTAGA
Orf19.6898.1 rt	FOR: TGAGCAAGTTCAGGAACGAAT<br><br>REV: AGTAGGATGAGCTTGACGTAATAATG
HEM15 rt	FOR: AGATTCGTATCCTGCTGAAGTG<br><br>REV: TCTGCACGCTTTATCTTGTGT
ECE1 rt	FOR: GAGATGGCGTTCCAGATGTT<br><br>REV: TACTGAGCCGGCATCTCTTT
ADAEC rt	FOR: TGCCATCATCAGCTGCTCCTGC<br><br>REV: CGGTGGTGTCTTCATCTGCGCC
PDX1 rt	FOR: TAACTTCAAACTTGGGTGAAGATAT<br><br>REV: GTTCATCAAAACTATCTGCTGAAAC
Orf19.805 rt	FOR: TGATAAAATGGGAGAGGTCATCT<br><br>REV: TCTTCAGGGAACGGCACT

YNK1 rt	FOR: TGGTTCAACCAACTGAATCTTTAT<br><br>REV: TGTTAGCAGATTCAACAGAATCAG
SSK1 rt<br><br>DFI1 rt	FOR: ATGGCCAAGAAGCAATAGATAA<br>REV: AGCATTAGTCTTATCCACAGAAGAA<br>FOR: TACATCAAGTCCTGCAACAACA<br><br>REV: AAGTCCATCCACCTTCATAATCA
Orf19.3586 rt	FOR: GATCAGAAGGTACCACAGTTACTGA<br><br>REV: TGGTCCTGACGGTCTCAGA
Orf19.4715 rt	FOR: AGAGCTGCTGCTATAGTAAGTGTTG<br><br>REV: GATTCTGACTTGACAGCAGCA
FUN12 rt	FOR: TGGAAGCTCGTCGTAAAGAGT<br><br>REV: AGTTGGTTGAGCAGAAGATGGA
ACT1 rt	FOR: GGGTAGGGTGGGAAAACTTCA<br><br>REV: TTGAAACCACTGCCGACAGA
RTA4 chip	FOR: TGGGTTGGACTTTGCACTTT<br><br>REV: AGAATTGGGCAACTATCGGAT
PGA17 chip	FOR: GTTTTCATACCCCTGGAAGAACA<br><br>REV: TGGTGTGACAACTTGCTGTG
Orf19.4601 chip	FOR: TTCCGGGGAAAAGAAAATCT<br><br>REV: AAGATCCAGCAAGGCAAGAA
GSC1 chip	FOR: TTCATTAACACGGTCGCGAG<br><br>REV: TGCCCTCCTTTCCTTTGGAT
PDC12 chip	FOR: ACACAAACAGTTACAAACAAGCA<br><br>REV: GCCGAGTATGCTGCATTCAA
UTP22 chip	FOR: TCACGTGAATCCTGCATATT<br><br>REV: TCTTGGAATTGAACGGACAA
Orf19.7063 chip	FOR: TGTTCTTCTCAACTTGTATTCTGGT<br><br>REV: TGCGAGAACTTGTGGAAACC
ARO80 chip	FOR: GCTATCGACGGTAATTGAACAAT<br><br>REV: GTGGACTCGGCAACTTCTG

NOP14 chip	FOR: CGCTTGTCTTTTGTGTGGGA<br><br>REV: GGTAATGATGTTGTTAATTGAAAGAG
ECE1 chip	FOR: GTCTCACACGGTTAGAAGTCA (PF4)<br><br>REV: GGGCGGGCTTTACAAGTTTA (PR4)
ECE1 chip	FOR: TTTAGTCGTACTTGTCATGCT (PF5)<br><br>REV: ACGAATGGAAAATAGTTGGTAGT (PR5)
ADAE chip	FOR: CAATTTAGTTTCGCTTAAGACTG (PF4)<br><br>REV: GTGCAGATACCGTATGTCTCA (PR4)
ADAEC chip	FOR: TCAATTGGAAACGACGGA (PF5)<br><br>REV: AGCTTCGCTAGAATAAACCATCT (PR5)
DFI1 chip	FOR: CTGGCAAAATGGGATGTAAT (PF4)<br><br>REV: TTGACAAATTATTGACGCAAGT (PR4)
DFI1 ch5 chip	FOR: TTCTCTCTCATTCACTCACTCAC (PF5)<br><br>REV: AGGATGGTATAATAAATAGTAAAGGTGA (PR5)
PDX1 chip	FOR: CTGTACGCCAAAGTTGTTG (PF4)<br><br>REV: AAGTCCAGCTCTGAATAACTATACA (PR4)
PDX1 chip	FOR: TGATAGTTTGACATTTTGACAATT (PF5)<br><br>REV: GAAAGCTGCCCTAAGCAT (PR5)
CHK1 chip	FOR: ACCTTCATGAATTATCATTAGTGAA<br><br>REV: TGCAGGTTCGCTTGAATTA
Orf19.1698 chip	FOR: CAGGGCTTCTTAATAGTAGATCG<br><br>REV: ACTTTCATCTCGTGCAGTCAT
CDR2 chip	FOR: TCAAAGCCTCTATTATGAATACTAGTAG<br><br>REV: CGTCTAGCTGTTGCGACA
PMT1 chip	FOR: TTACAACAACAACAACAACCAC<br><br>REV: ACCGGTTTCTTTGCCATA
HEM15 chip	FOR: TCGCTGCCATTGGTTAAT<br>REV: ACTTATTAGTCACGAACGTGTCA
ROD1 chip	FOR: ATAGAGATCTGGAATCTGAAATCTA<br><br>REV: CATTCATCACATCACATCGA
Orf19.6898.1 chip	FOR: GTGTTATGTTAAAATAAATCGCCA

	REV: TACTGGTTACTGGTTGCTTGTTA
ADH1 chip	FOR: GCAACAGCAGGTGCCACC<br><br>REV: GGCTTTTTGAGTTTTTGG
ACT1 chip	FOR: GTTTGTTGTTTATAGTCG<br><br>REV: TATTTTTTTAATATTAATATCG
CDR1 chip	FOR: GTTCGATACTGTTAGTAATGTTC<br><br>REV: TGAGGGCGGCTGTGTGT
RTA3 chip	FOR: TCAGCATTTAGAGAGTGATTTG<br><br>REV: CTGCTGCAACCACATTTC
MDR1 chip	FOR: TCCTAAAATGAAAATTGATGTA<br><br>REV: TGACTCATCACACGAACAA

rt: primers used in RT-PCR (Reverse transcription PCR)

chip: primers used in ChIP (chromatin immunoprecipitation)
